# A smart acoustic textile for health monitoring

**DOI:** 10.1038/s41928-025-01386-2

**Published:** 2025-05-19

**Authors:** Yingqiang Wang, Chaochao Sun, Daniel Ahmed

**Affiliations:** Acoustic Robotic Systems Lab (ARSL), Institute of Robotics and Intelligent Systems, ETH Zurich, Rüschlikon, Switzerland

**Keywords:** Biomedical engineering, Mechanical engineering, Electrical and electronic engineering

## Abstract

Wearable electronics, such as smart textiles, are of potential use in healthcare monitoring, human–machine interfaces and environmental analysis. However, the scalability and reliability of the technology is restricted due to challenges related to rapid material degradation, potential toxicity, high production costs and heavy computational workload. Here we report an acoustic-based smart textile technology. The approach, which we term SonoTextiles, uses piezoelectric transducers that are mounted at both ends of glass microfibres and act as transmitters and receivers of acoustic waves. The flexible glass microfibres act as acoustic waveguides and are embedded into the textile substrate, providing precise sensing by measuring wave propagation and energy loss along the fibre in response to stimuli such as touch and bending. We also use acoustic frequency selectivity and frequency-domain signal processing algorithms to enhance computational efficiency. Our acoustic textile is breathable, durable and stable under thermal fluctuations, and we show that it can be used in distributed tactile sensing, hand gesture recognition and respiratory rate monitoring.

## Abstract (web summary)

Acoustic fibre waveguides that are woven into a textile substrate can be used to create a smart textile that can sense touch and bending, with applications in distributed tactile sensing, hand gesture recognition and respiratory rate monitoring.

## Main

Wearable electronics, such as smart textiles sensors, can enhance real-time interaction and perception across various environments^1–8^. Textile sensors can, in particular, be used for in situ healthcare monitoring, human–machine interfaces and environmental analysis^9–12^. Such textiles involve the integration of sensing technologies with fabric bases^9,13^. A variety of functional materials can be used to create these systems, including electromagnetic^14^, piezoelectric^15^, triboelectric^16^ and magnetoelastic^17^ materials, which are fabricated within the fibres through manufacturing techniques such as coating, spinning, printing and thermal drawing^18–23^. Acting as yarns, these functionalized fibres are spun into smart textiles by weaving, knitting or other structuring means^9^.

When incorporated into everyday clothing, the textiles can detect physiological and environmental factors such as pressure, strain, humidity, temperature and sound, and they should, ideally, be breathable, flexible and lightweight^24–28^. Smart textiles can be designed to harvest energy from environmental sources^12^ and physical activities (such as body movements)^14^ to power sensors and other electronic components^24,29^. These textiles can also be designed to emit optical or acoustic signals, providing real-time interaction between the wearer and external systems^10,12^.

For smart textiles to be of wider practical value, they must be washable, robust, safe, non-toxic and cost-effective^9,30^. However, the functional materials typically suffer from rapid degradation, which complicates their integration into garments that undergo frequent friction and washing^9,31^. Furthermore, complex manufacturing processes and the use of rare raw materials leads to high costs^9,12^. The use of materials such as graphene nanoribbons^28^, polypyrrole^27^ and barium titanate^12^ also creates potential health risks and requires extensive toxicity evaluations^32^, particularly for wearable applications targeted at children or pregnant individuals. Moreover, efficient in-situ computing is crucial for smart textiles, especially for array-based sensory systems that handle large data volumes^9,30^.

Acoustic technologies can offer non-invasive monitoring, biocompatibility, cost-effectiveness, versatility and straightforward integration^10,33–36^, and smart textiles based on acoustic technologies have previously been used in human–machine interfaces and biomonitoring. For example, piezocomposite single fibres woven into a shirt have been used to measure the direction of acoustic impulses and facilitate bidirectional communication between similar garments, enabling the auscultation of cardiac sound signals^10^. However, the approach involves complex fabrication processes to form the nanostructured piezoelectric composites and the fibres^10^. As an alternative, an all-nanofibre mechano-acoustic sensor based on a standalone thin film has been used for the continuous monitoring of mechano-acoustic heart signals^36^. The approach demonstrated high mechanical robustness and consistent performance, but functioned as a standalone thin film, without integration into textiles. A key advantage of acoustic sensors is their frequency selectivity^37,38^, which can provide a selective response^33,39^, channel frequency multiplexing^40^ and out-of-band noise suppression^41^.

In this article, we report an acoustic-based smart textile for human–machine interactions and healthcare monitoring. The technology, which we term SonoTextiles, uses piezoelectric transducers (PZTs) mounted at both ends of glass microfibres to transmit and receive acoustic waves. The flexible glass microfibres act as acoustic waveguides and are embedded into a textile substrate to provide guided propagation of acoustic waves. The textile detects external stimuli in real time by measuring acoustic wave attenuation and energy loss within the fibre waveguide. We use acoustic frequency selectivity and frequency-domain signal processing algorithms to reduce system complexity and improve computational efficiency. We also incorporate frequency division multiple access (FDMA), which is a technique with dedicated frequency band allocation that is widely used in telecommunications^42^, to reduce the number of sensors, electrical connections and wires required for distributed sensing applications. We show that the smart textile can be used for tactile sensing, hand gesture recognition and respiratory rate monitoring. Our approach is based on a cost-effective design that avoids complex manufacturing processes and advanced functional materials. The system is also breathable, durable, thermally stable and safe.

## Acoustic waveguide for smart textile sensing

**Fig. 1:**
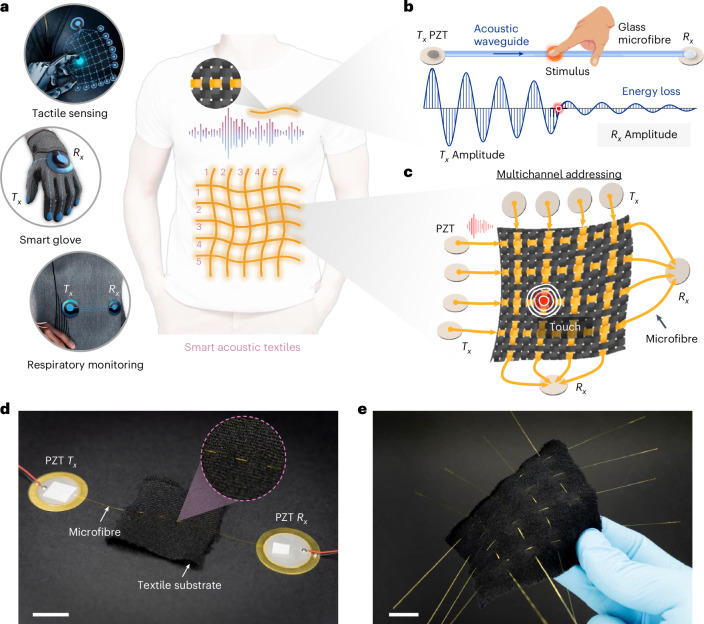
Smart textiles using glass-fibre acoustic waveguides. **a**, Concept of the proposed smart acoustic textiles. The acoustic waves transmitted and received by the PZT transducers will propagate along the microfibre waveguide woven in the textile, completing various wearable sensing and interactive tasks. **b**, Illustration of a basic SISO system. The acoustic wave generated by the transmitting PZT (${T}_{x}$) is transmitted through the waveguide of the glass microfibre to the receiving PZT (${R}_{x}$). Acoustic waves in glass fibre experience natural attenuation due to contact with the yarn. When external stimuli, such as touch or bending occur, this contact is increased and brings additional acoustic energy loss, as reflected by the ${R}_{x}$ amplitude. **c**, Illustration of a tactile sensing array using a MISO pattern along both weft (latitude) and warp (longitude). The ${R}_{x}$ can determine which ${T}_{x}$ the acoustic wave is coming from based on the assigned frequency and can detect the coordinate being touched. See Fig. 3 for more information. **d**, Image of a SISO system. The glass fibre is woven into a textile substrate. **e**, Image of the sensing array. Four warp fibres and four weft fibres form a 4 × 4 textile sensing array with a total of 16 touchpoints. Scale bars, 10 mm. Credit: man in **a**, halayalex, Freepik.com; hand icon in **b**, Freepik.com.

A waveguide is a device designed to direct waves along a predetermined path, effectively confining them to minimize energy loss over distances^43^. SonoTextiles operate on the principle of propagating acoustic waves, predominantly Lamb waves, within glass microfibre waveguides^44,45^. The acoustic energy loss during this propagation is sensitive to external stimuli, allowing the textile to function effectively as a sensing platform. The smart acoustic textiles distribute microfibre waveguides on everyday clothing as a wearable electronic device to complete various sensing and interactive applications, such as tactile sensing, smart gloves and respiratory monitoring (Fig. 1a). To demonstrate the mechanism, we first configured our system as a single-input single-output (SISO) system (Fig. 1b). The SISO system primarily comprises a transmitting PZT ($${T}_{x}$$), a receiving PZT ($${R}_{x})$$, a glass microfibre and the textile substrate. The $${T}_{x}$$ performs electroacoustic conversion, whereas the $${R}_{x}$$ performs acoustoelectric conversion (see Methods for more details). The glass microfibre, woven into the textile substrate, is connected at one end to the $${T}_{x}$$ PZT and at the other to the $${R}_{x}$$ PZT, serving as the waveguide for $${T}_{x}$$-generated acoustic waves. When external stimuli, such as applied pressing forces from physical touch or contact, are applied to the smart textile, any resultant changes in the amplitude or energy of the received acoustic signal are detected by the $${R}_{x}$$ PZT, as shown by the decay in acoustic wave amplitude in Fig. 1b. Specifically, acoustic waves in the microfibre waveguide attenuate and undergo significant energy loss due to the contact between glass microfibres and yarns or skin. Building on this foundational principle, we have developed various systems with specific functions, such as the fibre-array tactile sensing interface illustrated in Fig. 1c. The thin and flexible glass microfibres are integrated into the textile substrate along both warp and weft directions, forming a distributed tactile sensing interface where each intersection serves as an addressable touchpoint. Based on various applications, the layout of the glass-fibre waveguides can be customized with unique features to create diverse smart textile systems. Figure 1d shows an image of the SISO system, where the $${T}_{x}$$ and $${R}_{x}$$ PZTs can be seen, as well as the glass microfibre connected to the PZTs and woven into the black textile substrate (see Supplementary Fig. 1 for more details). Figure 1e shows a fibre-array tactile sensing interface that features 16 touchpoints arranged in a 4 × 4 grid.

**Fig. 2:**
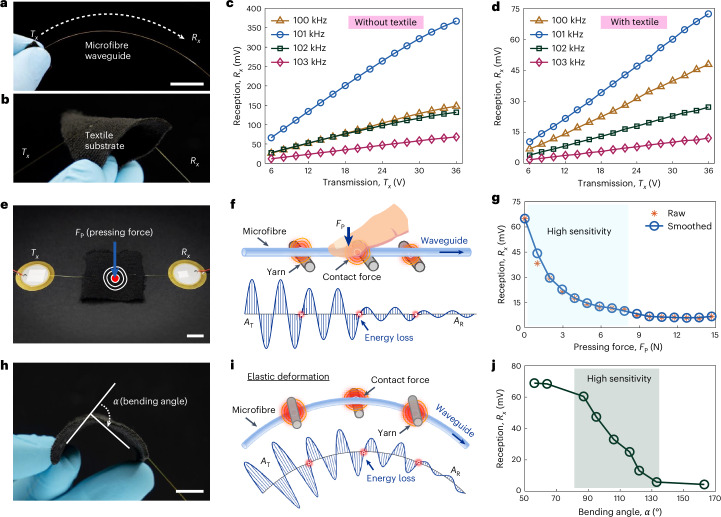
Characterization of the smart acoustic textiles. **a**, Image of the glass microfibre. The natural drooping state of the fibre illustrates its flexibility and elasticity. The fibre acts as the acoustic waveguide between the ${T}_{x}$ and ${R}_{x}$ PZTs. **b**, Image of a SISO system with the fibre woven into a black textile substrate. The substrate is composed of a two-thirds cotton and one-third polyester blend. The smart acoustic textile is flexible. **c**, Plot of ${R}_{x}$ reception versus ${T}_{x}$ transmission peak-to-peak amplitude in the SISO system (without textile substrate) at different signal frequencies. **d**, Plot of ${R}_{x}$ reception versus ${T}_{x}$ transmission peak-to-peak amplitude in the SISO system (with textile substrate) at different signal frequencies. **e**, Illustration of the application of external pressing force (*F*_P_) by a finger on the SISO system. **f**, Principle analysis of acoustic energy loss caused by *F*_P_. The touch not only causes energy dissipation at the contact point between the finger and fibre, but also increases the contact stress between fibre and yarns, resulting in more energy loss, as reflected by the ${T}_{x}$ amplitude (*A*_T_) and ${R}_{x}$ amplitude (*A*_R_). **g**, Plot of ${R}_{x}$ reception peak-to-peak amplitude versus *F*_P_. The data has been smoothed with a moving average to highlight the trend. The shaded region indicates higher sensitivity during the initial increase in *F*_P_. **h**, Illustration of bending angle perception by the SISO system. **i**, Principle analysis of acoustic energy loss caused by the bending. The elastic deformation of glass microfibre increases contact forces between the fibre and yarns, leading to significant acoustic energy loss. **j**, Plot of ${R}_{x}$ reception peak-to-peak amplitude versus bending angle (*α*). The shaded region indicates higher sensitivity within this bending angle range. The data in **c,** **d**, **g** and **j** were collected under static conditions. The oscilloscope used to measure data in **c,** **g** and **j** has a small error (8-bit amplitude resolution, that is, 1/256 of the full scale). Scale bars, 10 mm. Credit: finger icon in **f**, Freepik.com.

To elucidate the mechanism and functionalities, we conducted tests using the SISO system as our initial framework. We first evaluated the propagation of sinusoidal acoustic waves at different frequencies (Supplementary Fig. 2). The evaluation is divided into two cases: using the SISO system of a single fibre without textile (Fig. 2a); and using the SISO system with a textile substrate (Fig. 2b). Our results showed that the ${T}_{x}$ and ${R}_{x}$ signal amplitudes were directly proportional across the tested frequencies of 100, 101, 102 and 103 kHz, suggesting the feasibility and robustness of the proposed microfibre waveguide for acoustic propagation within a wide frequency band (Fig. 2c,d). These frequencies were chosen based on the impedance characterization results of the selected PZT transducers for better piezoelectric performance (Supplementary Note 1 and Supplementary Fig. 3). Because the contact between the fibre and yarn causes more acoustic energy loss, the ${R}_{x}$ amplitudes in Fig. 2d are significantly lower than those in Fig. 2c. However, both show consistent and reliable input–output characteristics. Furthermore, when holding the ${T}_{x}$ amplitude constant, we observed that a frequency of 101 kHz produced a higher ${R}_{x}$ amplitude compared with the other selected frequencies. We attribute this to the resonance peaks in the PZT impedance characterization results. When multiple frequencies are used simultaneously, different ${T}_{x}$ amplitudes can be adjusted through excitation voltages to ensure consistent ${R}_{x}$ amplitude between the channels. Moreover, to further verify the robustness of the acoustic wave propagation mechanism in the fibre waveguide, we assessed the input–output characteristics of the SISO system under various weaving conditions on the same black textile substrate (Supplementary Fig. 4).

External forces, including pressing and bending, are common stimuli to which smart textiles must respond. To explore this, we initially investigated the impact of external pressing force on the SISO system by applying a single finger-touch, (Fig. 2e). The mechanism of pressing force sensing is shown in Fig. 2f. Contact between the finger and the glass fibre results in acoustic energy dissipation. In addition, the contact between the fibres and the yarns increases with touch, causing further energy loss. As shown in Fig. 2g, we observed that the ${R}_{x}$ peak-to-peak amplitude decreased from 65.0 to 6.7 mV (that is, an energy loss of 19.7 dB, see Methods for the calculation) as the applied pressing force increased from 0 to 14.7 N (Fig. 2e), demonstrating the sensitivity of the SISO system to external pressing forces (see Supplementary Fig. 5 for the experimental set-up). This SISO system notably exhibited exponential decay in amplitude during the initial phase of increasing pressing force, as indicated by the steep downward slope of the curve in Fig. 2g. As the pressing force increased from 0 to 7.8 N, the ${R}_{x}$ peak-to-peak amplitude decreased from 65.0 to 11.0 mV, resulting in an energy loss of 15.5 dB. By contrast, when the pressing force increased from 7.8 to 14.7 N, the ${R}_{x}$ peak-to-peak amplitude further dropped from 11.0 to 6.7 mV, with an energy loss of 4.3 dB. This shows that the textiles are highly sensitive to small-value external stimuli.

The capacity of a flexible wearable system to detect self-bending enhancement can be useful in monitoring human physiological states. Figure 2h demonstrates the SISO textile system’s detection of bending angles. Bending-induced elastic deformation and the force exerted on the glass microfibre enhance the contact stress between the fibre and yarns, thereby increasing acoustic energy loss, as illustrated by the schematic analysis in Fig. 2i. The relationship between bending angle and ${R}_{x}$ signal amplitude is shown in Fig. 2j, demonstrating the SISO system’s ability to detect its self-bending. From the change in the slope of the curve in Fig. 2j, we observed that the SISO system is particularly sensitive between bending angles of 90° and 120°, where the ${R}_{x}$ signal experienced an energy loss of 13.2 dB as the bending angle increased from 87° to 122°. The ability to sense external stimuli enables the expansion of the SISO system to include more complex acoustic textiles such as fibre-array tactile interfaces, smart gloves and physiological monitoring systems.

## Fibre-array tactile sensing interfaces

The sensing capability of the foremost SISO system is limited to a one-dimensional glass-fibre path and does not meet the two-dimensional requirements needed for many wearable devices. A viable solution to overcome this drawback involves arranging multiple independent SISO systems into a two-dimensional textile matrix. However, this approach necessitates a substantial increase in the number of PZTs, both as ${T}_{x}$ and ${R}_{x}$, increasing both system complexity and cost. To overcome this, our design integrates a multi-input single-output (MISO) system architecture with multiple inputs (${T}_{x}$ PZTs) and a single output (${R}_{x}$ PZT), aligned along the warp and weft directions (Fig. 1c). The MISO configuration allows multiple acoustic waveguides to be distributed across the textile substrate in both longitude and latitude directions, creating a two-dimensional sensing interface. Not only does this enable basic sensing of external stimuli using a single waveguide but it also facilitates detailed analysis of data across both axes to accurately pinpoint stimulation sites within the two-dimensional sensory array. However, a challenge in the MISO architecture arises when the signal at the ${R}_{x}$ PZT comprises a superposition of multiple ${T}_{x}$ signals, resulting in temporal overlap and interference. Addressing this issue requires the development of an alternative and suitable signal processing strategy.

FDMA, which is a foundational technology in telecommunications, assigns unique frequencies to multiple channels to enable simultaneous transmission without interchannel interference^42^. As we show in Fig. 2c,d, our smart textile has stable input–output performance at 100, 101, 102 and 103 kHz, which can be used for FMDA addressing. In the proposed MISO architecture, FDMA allocates those distinct frequencies to each glass-fibre waveguide in both the warp and weft directions. This implementation of FDMA enables fast Fourier transform (FFT)-based array addressing computations in the frequency domain, resolving the issue of multichannel interference of the superimposed signal in the time domain, while only using one ${R}_{x}$ PZT receiving signal. Each frequency is characterized by specific power or amplitude levels across the frequency spectrum, evaluated through FFT-based computations, in the reception end of the warp and weft ${R}_{x}$ PZTs.

When exposed to external stimuli, such as touch, a fibre waveguide in the affected channel shows an acoustic energy loss, which is typically greater than 6 dB, corresponding to a 50% reduction in amplitude compared with unaffected channels. To validate our approach, we conducted a MATLAB based simulation on a four-channel MISO system (Supplementary Fig. 6). This simulation employed short-time Fourier transform, which is essentially FFT applied with a window function, to provide detailed time–frequency analyses. We modelled both the stationary state and scenarios where the second channel experienced energy losses of 6 and 18 dB due to external stimuli. The lower peaks in single-sided amplitude spectra, along with diminished band brightness in time–frequency spectrograms, conclusively identified the second channel (101 kHz) as affected by external stimuli, demonstrating the efficacy of FDMA addressing (Supplementary Fig. 6).

**Fig. 3:**
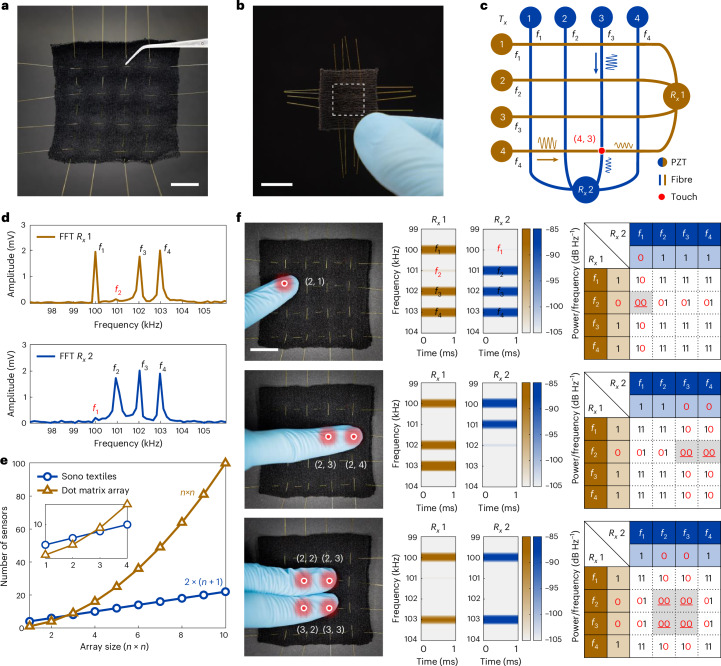
Acoustic fibre array as a wearable tactile sensing interface. **a**, Image of the fibre-array acoustic textile with 16 touchpoints in a 4 × 4 grid. **b**, Image of a smaller fibre-array acoustic textile, also with 16 touchpoints. Its core array sensing area measures only approximately 10 mm by 10 mm, which is about the size of a human fingernail, as indicated by the dashed box. **c**, Schematic diagram of the 4 × 4 fibre-array acoustic textile system. Touch or a pressing force will affect acoustic wave propagation in specific channels along the warp and weft directions. The pressed point, indicated by the red dot, is at coordinates (4, 3). **d**, FFT-obtained single-sided amplitude spectra of ${R}_{x}$ 1 and ${R}_{x}$ 2 when the point with coordinates (2, 1) is pressed. The frequency *f*_2_ along the weft direction and the frequency *f*_1_ along the warp direction are blocked, and hence the peaks corresponding to *f*_2_ (${R}_{x}$ 1) and *f*_1_ (${R}_{x}$ 2) are nearly absent. **e**, Comparison plots of the number of sensors versus the array size between the MISO SonoTextiles array and the dot matrix array for an array with *n* × *n* touchpoints. **f**, Images, time–frequency spectrograms and FDMA addressing results for three different tactile sensing cases involving the 4 × 4 array system. Images on the left depict the number of pressing points applied: one, two and four, respectively. Corresponding spectrograms are shown in the middle column. The right column displays FDMA addressing matrices indicating the tactile perception results. Frequencies with normal amplitudes are designated with 1, whereas frequencies with significant energy loss (greater than 6 dB) are indicated by 0. When both the weft and warp directions exhibit 0, represented as 00 in the matrix, the corresponding coordinates signify the perceived touchpoint by this tactile sensing array. Scale bars, 10 mm.

We developed a wearable fibre-array tactile sensing system based on the FDMA-based MISO architecture. We integrated four glass fibres into a black textile substrate along both the warp and weft, forming a 4 × 4 sensing array with 16 touchpoints (Fig. 3a). This tactile sensing textile retains the substrate’s inherent flexibility and breathability (Supplementary Videos 1 and 2). Furthermore, we configured a 4 × 4 array within a 10 mm by 10 mm square, (Fig. 3b). This compact miniature array, approximately the size of a human fingernail, addresses the challenges associated with small-scale wearable devices.

Figure 3c shows a schematic of the 4 × 4 array, with MISO systems featuring four waveguide channels per axis. Each axis features four waveguide channels, each assigned a unique frequency— *f*_1_, *f*_2_, *f*_3_ and *f*_4_ at 100, 101, 102 and 103 kHz, respectively, using the same PZT transducers as in Fig. 2. Computations for the signals received by ${R}_{x}$ 1 and ${R}_{x}$ 2 are conducted separately, enabling simultaneous use of all frequencies across both directions. FDMA addressing is achieved by evaluating the amplitude of each frequency received by the two ${R}_{x}$ PZTs in the frequency domain.

External stimuli, such as touch or a pressing force, can disrupt the propagation of acoustic waves within specific channels and potentially stop them entirely. Figure 3d shows the FFT-derived single-sided amplitude spectra of ${R}_{x}$ 1 and ${R}_{x}$ 2, resulting from a pressing force applied at coordinates (2, 1). The pressing force almost completely obstructed the waveguide in the weft (${R}_{x}$ 1) channel 2 at frequency 101 kHz (*f*_2_), resulting in the near absence of the corresponding 101 kHz peak in the spectrum with an acoustic energy loss of 21.9 dB. Similarly, the same pressing force affected wave propagation in the warp (${R}_{x}$ 2) channel 1, leading to the absence of the corresponding spectrum peak (*f*_1_) with an acoustic energy loss of 19.6 dB. Analysis of data from ${R}_{x}$ 1 and ${R}_{x}$ 2 enabled the identification of pronounced acoustic energy loss in weft channel 2 and warp channel 1, and pinpointed the touched coordinate as (2, 1). This confirms the implementation of a fundamental FDMA addressing scheme within the tactile perception interface of this array.

Unlike traditional dot matrix arrays, which necessitate data computation for each sensor at every touchpoint, this FDMA-based MISO architecture simplifies processing by requiring frequency-domain computation for signals from just the two ${R}_{x}$ PZTs, enhances data processing efficiency. In addition, MISO systems can streamline the array system by using fewer sensors, which thus reduces the need for extensive electrical connections and wiring. Figure 3e shows comparison plots of the number of sensors versus the array size between the MISO SonoTextiles array and the dot matrix array. For an array with *n* × *n* touchpoints, the SonoTextile needs 2 × (*n* + 1) sensors, whereas the traditional dot matrix approach needs *n* × *n* sensors. As shown in the inset of Fig. 3e, when *n* exceeds three, the number of sensors needed in the dot matrix array is greater than that in SonoTextiles. Moreover, it is foreseeable that in larger-scale arrays, as *n* increases, the difference will become increasingly pronounced because *n* × *n* rises exponentially, demonstrating the advantages of the smart acoustic textiles in terms of simplifying system complexity by employing fewer sensors and thus reducing electric connections and wires (see Supplementary Fig. 7 for details).

We subsequently conducted comprehensive tactile perception experiments using the 4 × 4 array textile. Figure 3f displays images, time–frequency spectrograms and FDMA addressing results from three different tactile sensing scenarios involving both single and multiple pressed touchpoints. Pressing at (2, 1) yielded spectrograms for ${R}_{x}$ 1 and ${R}_{x}$ 2 that were consistent with those shown in Fig. 3d,e, respectively, with significant energy loss at 101 kHz (weft) and 100 kHz (warp). Similarly, pressing simultaneously at points (2, 3) and (2, 4), as well as at four points (2, 2), (2, 3), (3, 2) and (3, 3), produced corresponding spectrograms and FDMA addressing matrices that accurately detected all touched coordinates. The full results of these touch perception experiments on the 4 × 4 array are shown in Supplementary Video 3.

## SonoGloves for hand gesture recognition

**Fig. 4:**
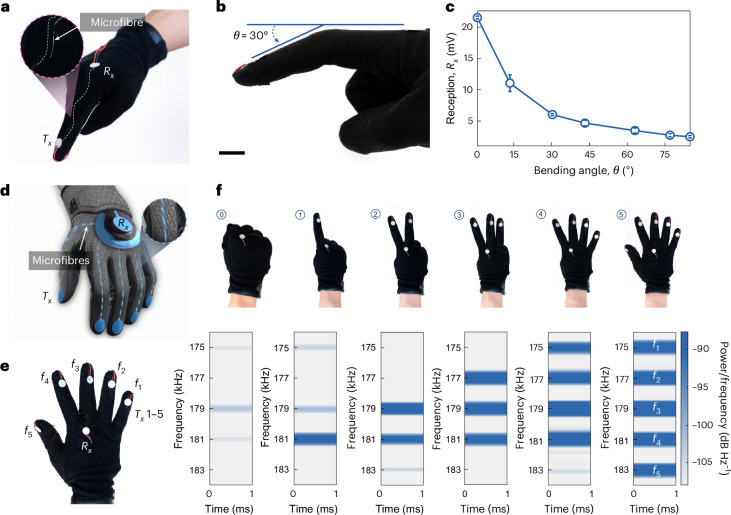
Smart gloves for hand gesture recognition. **a**, Image of a smart glove using a SISO acoustic waveguide. The single glass fibre is woven into the textile substrate along the index finger. The white circular components are ${T}_{x}$ and ${R}_{x}$ PZTs for acoustic wave transmission and reception. **b**, Schematic representation of index finger bending sensing using the smart glove. **c**, Plot of finger bending angle (*θ*) versus ${R}_{x}$ peak-to-peak amplitude of the smart glove, showing a correlation. Data are presented as mean ± s.d. (*n* = 5) using the same glove, with uncertainty primarily arising from slight, unavoidable finger movements during repeated measurements. **d**, Illustration of gloves equipped with a MISO system. **e**, Image of the smart glove. Integrated glass microfibres run along the five fingers, and the five ${T}_{x}$ PZT elements are allocated different operating frequencies. **f**, Images of hand gestures performed with the smart glove for gesture recognition applications, along with the corresponding time–frequency spectrograms of ${R}_{x}$ received signals. The ${R}_{x}$ spectrograms accurately indicate the six gestures. Scale bar, 10 mm.

The hand, an integral sensory and executive body part, continues to be a key focus in the research of wearable technologies. Here we developed the SonoGloves that employs acoustic smart textiles to detect finger bending and facilitate hand gesture recognition. A basic SISO version of the glove is illustrated in Fig. 4a, with a more detailed schematic presented in Supplementary Fig. 8. A single glass microfibre is integrated along the index finger of the glove. The glove is equipped with ${T}_{x}$ and ${R}_{x}$ PZTs, enabling the transmission and reception of acoustic waves.

The glove can perceive index finger bending (Fig. 4b,c). Notably, when the finger bends, the fibre waveguide undergoes significant acoustic energy loss due to both self-bending (Fig. 2i) and the contact pressing force between the finger joint and glove. Figure 4c shows the relationship between the bending angle of the finger and the ${R}_{x}$ peak-to-peak amplitude. Maintaining a constant ${T}_{x}$ signal amplitude, we show that as the bending angle increases from 0 to 85°, the acoustic wave attenuates and decays exponentially, eventually leading to near-complete blockage of wave propagation and an acoustic energy loss of 18.8 dB.

Further advancing our technological suite, we introduced the gloves in a MISO system architecture (Fig. 4d,e and Supplementary Fig. 8). This variant integrates glass microfibres extending across all five fingers, each linked to one of five identical ${T}_{x}$ PZTs, which collectively converge at a singular ${R}_{x}$ PZT. The five ${T}_{x}$ PZTs are set to operate at distinct frequencies, enabling FFT-based FDMA addressing in the frequency domain: 175 kHz for the smallest finger, 177 kHz for the ring finger, 179 kHz for the middle finger, 181 kHz for the index finger and 183 kHz for the thumb. The operating frequencies here are higher than those used earlier, as a smaller-sized PZT transducer was employed to fit the dimensions of gloves (see Methods and Supplementary Note 1 for details). This design ensures the glove’s flexibility, preserving the natural dynamics of hand movement for enhanced functionality.

With its MISO architecture, our acoustic smart glove can be used for interactive wearable applications, such as hand gesture recognition and potential sign-to-speech translation. We tested the glove’s capability to discern numerical gestures from zero to five (Fig. 4f). For example, in the gesture for ‘1’, the index finger is extended while the other four fingers remain clenched and wave propagation occurs exclusively through the index finger’s channel. The corresponding time–frequency spectrogram, marked by a distinct band at 181 kHz specific to the index finger, is shown in Fig. 4f. Similarly, for the gesture representing ‘2’, only the middle and index fingers are extended, facilitating wave propagation through these specific channels, as evidenced by two bands at 179 and 181 kHz in the spectrogram. Gestures for ‘0’, ‘3’, ‘4’ and ‘5’ accordingly show zero, three, four and five highlighted bands in their respective time–frequency spectrograms (Fig. 4f), each aligning with the number of extended fingers.

## Physiological monitoring

**Fig. 5:**
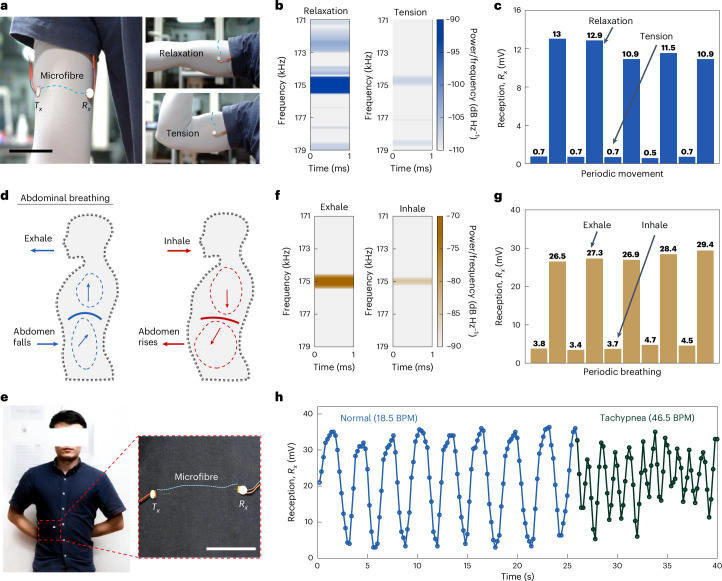
Physiological monitoring applications of the acoustic textile system. **a**, Illustration of muscle state monitoring using a SISO system. A fibre woven into a close-fitting garment can detect muscle relaxation and tension states. **b**, Time–frequency spectrograms of ${R}_{x}$ received signals under muscle relaxation and tension states. The clear distinction of states can effectively indicate muscle status in real time. **c**, Results from monitoring of the arm muscles in periodic motion. The periodic variation in ${R}_{x}$ peak-to-peak amplitude effectively monitors muscle state. **d**, Illustration of abdominal breathing in the human body. An acoustic textile can use the rhythmic rise and fall of the belly to achieve respiratory monitoring. **e**, Image showing integration of a SISO system into the abdominal area of a cloth shirt. **f**, Spectrograms of ${R}_{x}$ received signals under exhalation and inhalation states. **g**, Abdomen movement sensing data measured during periodic breathing. The variation in ${R}_{x}$ peak-to-peak amplitude identifies the rise and fall of the abdomen, corresponding to exhalation and inhalation states. **h**, Continuous monitoring and diagnosis of simulated abnormal respiratory rate (in BPM). The periodic variation in ${R}_{x}$ peak-to-peak amplitude effectively monitors respiratory rate and could provide timely alerts for abnormal conditions such as tachypnea. Scale bars, 100 mm.

Wearable flexible electronics, designed for prolonged contact with the body, are ideal for real-time physiological monitoring. Using our smart acoustic textile technology, we have developed systems for monitoring arm muscle activity and respiratory rates, essential for contexts such as patient rehabilitation and athletic training. For example, Fig. 5a demonstrates an application of a muscle state monitoring with a SISO acoustic textile system woven into a close-fitting garment. This set-up tracks the expansion and relaxation of arm muscles in real time. When muscles such as the biceps brachii tense, they partially expand, increasing the contact pressing force between the skin and the acoustic textile, thereby enhancing acoustic wave propagation loss. Conversely, when muscles relax, the contact pressing force decreases, leading to reduced propagation loss. Figure 5b depicts time–frequency spectrograms of the ${R}_{x}$ received signals during states of muscle relaxation and tension, revealing a 25.6-dB energy loss in tense states compared with relaxed ones. The difference between the two spectrograms enables the real-time determination of muscle status. Figure 5c demonstrates continuous monitoring of the biceps brachii in periodic motion, showing that the ${R}_{x}$ amplitude varies periodically, with an average acoustic energy change of 25.0 dB.

Another critical application is the real-time monitoring of respiratory rate, which is particularly vital for patients with respiratory ailments such as asthma. Figure 5d provides a schematic of abdominal breathing, which shows cyclical fluctuations with diaphragmatic movements during respiration. A SISO system, similar to the muscle monitoring set-up, leverages these rhythmic expansions and contractions for detailed respiratory monitoring, as detailed in Supplementary Fig. 9.

Our SISO system woven into the abdominal region of a form-fitting shirt, captures breathing patterns (Fig. 5e and Supplementary Video 4). Time–frequency spectrograms of the ${R}_{x}$ received signals, as illustrated in Fig. 5f, delineate the phases of exhalation and inhalation, revealing a 14.4-dB energy loss during inhalation. Furthermore, Fig. 5g highlights the monitoring of abdominal movements during periodic breathing, in which the ${R}_{x}$ amplitude exhibits periodic variations, with an average acoustic energy loss of 15.2 dB.

Figure 5h further demonstrates continuous monitoring and diagnosis of abnormal respiratory rates, such as tachypnea or bradypnea. The smart textile accurately responds to abdomen movements during breathing by capturing real-time variations in the ${R}_{x}$ signal amplitude. The periodicity of the ${R}_{x}$ signal amplitude curve directly reflects the observed respiratory rate (Fig. 5h). In a 40-second test, the subject maintained a normal respiratory rate of 18.5 breaths per minute (BPM) for the first 25 seconds, followed by accelerated breathing to simulate tachypnea at 46.5 BPM. This test showcases the SISO system’s capability to effectively monitor respiratory rates in real time when integrated into a garment. In addition, we noted that during simulated tachypnea (Fig. 5h), the fluctuation range of the ${R}_{x}$ amplitude was reduced compared with the normal state. Specifically, the average fluctuation range of the ${R}_{x}$ amplitude was 30.0 mV under normal conditions, whereas it decreased to just 18.1 mV during tachypnea. Rapid breathing results in less pronounced abdominal movements, recorded as a lower amplitude. These more subtle fluctuations could offer a valuable additional diagnostic tool for identifying respiratory abnormalities.

## Conclusions

We reported an acoustic-based smart textile platform for human–machine interactions and physiological monitoring. Our smart textile incorporates acoustic waveguides in microfibres with transmitting and receiving PZTs. The acoustic energy loss during acoustic wave propagation is sensitive to external stimuli, allowing the textile to function as a sensing platform. The acoustic sensing fibres can be incorporated with various textile substrates using different weaving conditions and shows consistent sensory performance and sensitivity (Supplementary Figs. 12, 14 and 15). Using the frequency selectivity of acoustic wave propagation, we incorporated FDMA to achieve distributed sensing systems. This simplifies the system architecture by reducing the number of sensors, electrical connections and wiring, and thus improves scalability. Furthermore, the use of FFT-based signal processing in the frequency domain enhances computational efficiency. Our smart textiles are also stable and durable when subjected to repeated stimuli and physical activities (Supplementary Fig. 13 and Supplementary Videos 5 and 6).

The acoustic smart textile technology platform is based on commercially available glass microfibres. The integration of multiple glass fibres into the textile substrate also does not affect the breathability, stability and flexibility of the textile substrate (Supplementary Fig. 10 and Supplementary Videos 1 and 2). The core components, including the PZTs and microfibres, help to enhance the overall affordability of the system, keeping the production cost within hundreds of dollars.

Future research is needed to overcome existing packaging limitations by integrating wearable power supplies, embedded data acquisition systems and wireless data transmission capabilities. The incorporation of sustainable, biodegradable materials could improve the environmental impact of the system. Haptic feedback could also be incorporated into the platform. Exploring diverse coupling methods between the glass microfibres and PZTs could optimize sensitivity and increase functionality and effectiveness. Future improvements could be achieved by tailoring the sensing properties of the fibres. By adjusting factors such as fibre length, diameter and material composition, we can control the mechanical response of the fibres and thus can tailor the system for specific applications with distinct working frequencies, thereby enhancing both the functionality and sensitivity of our sensing systems.

Our smart acoustic textile technology could be of use in a variety of fields. In physical therapy, smart gloves could be used to monitor finger movements and provide feedback to ensure that exercises are performed correctly. The smart gloves could also be used in stroke rehabilitation by tracking the recovery progress of patients through monitoring fine motor skills and hand movements. In addition, such gloves could potentially monitor disease progression of arthritis and aid in designing personalized exercise. In human–computer interactions, the gloves could be used in the gesture-based control of computers, smart devices and other electronic systems, providing an intuitive and hands-free method of interaction. In virtual and augmented reality environments, the gloves could enhance user experiences by offering precise hand and finger tracking, enabling more natural interactions. They could also be of use in gaming, robotics and teleoperation.

Distributed tactile sensing garments could be used by athletes to receive real-time feedback on their biomechanics during training or competitions. This data could then be used to optimize performance, prevent injuries and tailor training regimens to individual needs. Smart garments could also be used in assistive technology, providing alternative input methods for computers and other devices. The textiles could, in addition, provide tactile information to guide individuals with visual impairments, or to help to monitor and adjust posture for those with mobility issues. Future developments in artificial intelligence, 5G networks and the Internet of Things could expand the capabilities of our smart textiles, leading to opportunities in advanced computing, intelligent decision making and robust data connectivity^46–49^.

## Methods

### Acoustic energy loss

Acoustic energy loss, that is, the total energy loss during the propagation due to natural attenuation and external stimuli, is of key importance in the characterization of the smart acoustic textiles. For consistency, the acoustic energy loss, denoted by EL, is expressed in the following equation:

1 $${\rm{EL}}=20{\log }_{10}\left(\frac{{A}_{\rm{T}}}{{A}_{\rm{R}}}\right)$$

where *A*_T_ and *A*_R_ are the peak-to-peak amplitudes of the ${T}_{x}$ transmitted and ${R}_{x}$ received acoustic waves, respectively. The acoustic energy losses described in this article are all calculated using EL, with the unit being decibels (dB).

### PZTs

Commercially available PZTs made of piezoceramics are used in our experiments for the electroacoustic and acoustoelectric conversion. In the characterization of the SISO system and fibre-array tactile sensing experiments, we used the piezoelectric disc transducer (7BB-27-4L0, Murata Electronics) with a diameter of 27 mm and thickness of 0.5 mm for both the ${T}_{x}$ and ${R}_{x}$. In experiments with smart gloves and physiological monitoring applications, we selected the piezoelectric disc transducer (SMD10T2R111WL, STEMINC), which measured 10 mm in diameter and 2 mm in thickness.

The acoustic textiles operate at frequencies from 100 to 200 kHz, with PZTs achieving nanometre-scale oscillation. The vibration amplitude of transmitting PZTs depends primarily on the input voltage and piezoelectric coefficient, as described by the following equation^50,51^:

2 $${x}_{0}={d}_{33}\times V$$

where ${d}_{33}$ is the piezoelectric coefficient (which is approximately 600 × 10^−12^ m V^−1^ for PZT-5H) and $V$ is the applied voltage. The excitation voltages applied to the PZTs induce oscillations with nanometre-scale amplitudes. This high-frequency operation is both inaudible and imperceptible to the user, which minimizes the potential for discomfort^33^. In addition, based on our tests, these two types of PZT transducers do not exhibit significant heat generation under normal operating conditions, which makes them well suited for wearable applications (Supplementary Fig. 18).

### Glass microfibres

Commercially available glass fibres (FiberOptic P. & P. AG) made of silica (SiO_2_) with a diameter of 130 μm were used in our experiments, acting as the acoustic waveguide between the ${T}_{x}$ and ${R}_{x}$ PZTs. Glass fibre has a high Young’s modulus (72 GPa)^52^, which leads to high propagation speeds and low attenuation of acoustic waves in the waveguides^53^. In addition, glass fibre not only has good flexibility, but also exhibits excellent stability in high-temperature and corrosive environments, which makes it quite suitable for wearable devices.

### Textile substrates

Our fabric that we employed in this study was composed of a two-thirds cotton and one-third polyester blend (used for results shown in Figs. 2 and 3). The design criteria emphasized breathability, ensuring both comfort and functionality. Experimental validation confirmed that the acoustic textiles exhibit robustness and adaptability, demonstrating compatibility with a wide range of textile substrates varying in material composition and thickness (Supplementary Note 4 and Supplementary Figs. 14 and 15). Furthermore, the sensitivity and the effective propagation distance (that is, the wave travel distance along the fibre) are related to factors such as the textile substrate and weaving conditions (Supplementary Notes 3 and 5 and Supplementary Figs. 12 and 16).

### Fabrication method

The connection between glass fibres and PZTs serves as a critical interface for the propagation of acoustic waves between the microfibres and the PZTs. We employed copper foil tape (3M) to bond the PZTs to the glass microfibres. This method enables reusing the microfibres and PZTs during our research. However, a key challenge remains in achieving consistent and stable bonding strength, so further research is needed to achieve better bonding.

The glass fibres of the acoustic textiles are incorporated into the textile substrate through conventional weaving. We adopted the traditional manual weaving method in this work, using a hollow needle to sew the glass fibre into the textile. Although a digital sewing machine suitable for glass fibres can definitely provide more convenient and accurate weaving effects, we believe that manual weaving is sufficient for completing basic explorations of the smart textiles.

### Experimental set-up and data processing

Electronic function generators (AFG 1062, Tektronix) were employed to generate the predesigned waveforms which were finally connected to the ${T}_{x}$ PZTs. The observation and recording of the ${R}_{x}$ received signals were performed by a digital oscilloscope (TBS 2000, Tektronix), which could display the signal waveform and FFT results in real time and store the waveform with 8-bit amplitude resolution on a USB device. In the evaluations of the SISO acoustic textile (Fig. 2), a high-frequency signal amplifier (High Wave 3.2, Digitum-Elektronik) was connected between the function generator and the ${T}_{x}$ PZT to amplify the signal amplitude. This amplifier could provide an amplification gain of 15 times in the bridge mode. We note that the proposed acoustic textiles did not require this separate amplifier in the presented application scenarios, as the 20-*V*_PP_ output from the function generator was sufficient. In future work, we aim to integrate signal generation, analogue-to-digital and digital-to-analogue conversion, signal acquisition and other functionalities into compact wearable embedded systems.

The data processing of the ${R}_{x}$ received signal, including FFT-obtained single-sided amplitude spectra and time–frequency spectrograms, was primarily performed through postprocessing using computer software (MATLAB 2024a, Mathworks). Completing the digital signal processing tasks within wearable embedded systems is a key focus for future work.

### Ethical approval

This study was approved by the ETH Zurich Ethics Commission (number EK-2024-E-18). All procedures were conducted in accordance with the approved guidelines and regulations. Informed consent was obtained from the participant before the participant’s involvement in the study.

### Reporting summary

Further information on research design is available in the Nature Portfolio Reporting Summary linked to this article.

## Supplementary information

Supplementary Information Supplementary Notes 1–6, Figs. 1–18 and References.

Reporting Summary

Supplementary Video 1 Flexibility of the proposed smart acoustic textiles.

Supplementary Video 2 Breathability of the proposed smart acoustic textiles.

Supplementary Video 3 Acoustic fibre array as a wearable tactile sensing interface.

Supplementary Video 4 Continuous respiratory monitoring by a SISO SonoTextile system.

Supplementary Video 5 Stability of SonoTextiles after repeated external stimuli.

Supplementary Video 6 Durability of SonoTextiles under intense physical activities.

## Data Availability

The raw data underlying the analysis can be obtained from the corresponding author upon reasonable request.

## References

[1] Hu, H. et al. A wearable cardiac ultrasound imager. *Nature* **613**, 667–675 (2023). 36697864 10.1038/s41586-022-05498-z PMC9876798

[2] Zhang, B. et al. A three-dimensional liquid diode for soft, integrated permeable electronics. *Nature* **628**, 84–92 (2024). 38538792 10.1038/s41586-024-07161-1

[3] Huang, Y. et al. A skin-integrated multimodal haptic interface for immersive tactile feedback. *Nat. Electron.* **6**, 1020–1031 (2023).

[4] Zhuang, Q. et al. Permeable, three-dimensional integrated electronic skins with stretchable hybrid liquid metal solders. *Nat. Electron.* **7**, 598–609 (2024).

[5] Wang, M. et al. A wearable electrochemical biosensor for the monitoring of metabolites and nutrients. *Nat. Biomed. Eng.* **6**, 1225–1235 (2022). 35970928 10.1038/s41551-022-00916-z PMC10432133

[6] Lin, M. et al. A fully integrated wearable ultrasound system to monitor deep tissues in moving subjects. *Nat. Biotechnol.* **42**, 448–457 (2024). 37217752 10.1038/s41587-023-01800-0

[7] Xu, C. et al. A physicochemical-sensing electronic skin for stress response monitoring. *Nat. Electron.* **7**, 168–179 (2024). 38433871 10.1038/s41928-023-01116-6 PMC10906959

[8] Song, Y. et al. 3D-printed epifluidic electronic skin for machine learning-powered multimodal health surveillance. *Sci. Adv.* **9**, eadi6492 (2023). 37703361 10.1126/sciadv.adi6492 PMC10499321

[9] Libanori, A., Chen, G., Zhao, X., Zhou, Y. & Chen, J. Smart textiles for personalized healthcare. *Nat. Electron.* **5**, 142–156 (2022). 10.1038/s41928-022-00723-z PMC13309183 42367254

[10] Yan, W. et al. Single fibre enables acoustic fabrics via nanometre-scale vibrations. *Nature* **603**, 616–623 (2022). 35296860 10.1038/s41586-022-04476-9

[11] Kim, J., Campbell, A. S., de Ávila, B. E.-F. & Wang, J. Wearable biosensors for healthcare monitoring. *Nat. Biotechnol.* **37**, 389–406 (2019). 30804534 10.1038/s41587-019-0045-y PMC8183422

[12] Yang, W. et al. Single body-coupled fiber enables chipless textile electronics. *Science* **384**, 74–81 (2024). 38574120 10.1126/science.adk3755

[13] Rogers, J. A., Someya, T. & Huang, Y. Materials and mechanics for stretchable electronics. *Science* **327**, 1603–1607 (2010). 20339064 10.1126/science.1182383

[14] Lee, H. & Roh, J.-S. Wearable electromagnetic energy-harvesting textiles based on human walking. *Text. Res. J.* **89**, 2532–2541 (2019).

[15] Su, Y. et al. Muscle fibers inspired high-performance piezoelectric textiles for wearable physiological monitoring. *Adv. Funct. Mater.* **31**, 2010962 (2021).

[16] Chen, J. & Wang, Z. L. Reviving vibration energy harvesting and self-powered sensing by a triboelectric nanogenerator. *Joule* **1**, 480–521 (2017).

[17] Zhou, Y. et al. Giant magnetoelastic effect in soft systems for bioelectronics. *Nat. Mater.* **20**, 1670–1676 (2021). 34594013 10.1038/s41563-021-01093-1

[18] Park, M. et al. Highly stretchable electric circuits from a composite material of silver nanoparticles and elastomeric fibres. *Nat. Nanotechnol.* **7**, 803–809 (2012). 23178335 10.1038/nnano.2012.206

[19] Chatterjee, K. & Ghosh, T. K. 3D printing of textiles: potential roadmap to printing with fibers. *Adv. Mater.* **32**, 1902086 (2020). 10.1002/adma.201902086 31788860

[20] Barhoum, A. et al. Nanofibers as new-generation materials: from spinning and nano-spinning fabrication techniques to emerging applications. *Appl. Mater. Today* **17**, 1–35 (2019).

[21] Fan, W. et al. Machine-knitted washable sensor array textile for precise epidermal physiological signal monitoring. *Sci. Adv.* **6**, eaay2840 (2020). 32201720 10.1126/sciadv.aay2840 PMC7069695

[22] Kotz, F. et al. Fabrication of arbitrary three-dimensional suspended hollow microstructures in transparent fused silica glass. *Nat. Commun.* **10**, 1439 (2019). 30926801 10.1038/s41467-019-09497-z PMC6441035

[23] Yin, Y. et al. Flexible fluorescent metal-organic frameworks towards highly stable optical fibers and biocompatible cell platforms. *Sci. China Mater.* **66**, 1659–1669 (2023).

[24] Lee, J., Jeon, S., Seo, H., Lee, J. T. & Park, S. Fiber-based sensors and energy systems for wearable electronics. *Appl. Sci.* **11**, 531 (2021).

[25] Lee, J. et al. Conductive fiber-based ultrasensitive textile pressure sensor for wearable electronics. *Adv. Mater.* **27**, 2433–2439 (2015). 25692572 10.1002/adma.201500009

[26] Araromi, O. A. et al. Ultra-sensitive and resilient compliant strain gauges for soft machines. *Nature* **587**, 219–224 (2020). 33177670 10.1038/s41586-020-2892-6

[27] Hannigan, B. C., Cuthbert, T. J., Ahmadizadeh, C. & Menon, C. Distributed sensing along fibers for smart clothing. *Sci. Adv.* **10**, eadj9708 (2024). 38507488 10.1126/sciadv.adj9708 PMC10954209

[28] Tan, C. et al. A high performance wearable strain sensor with advanced thermal management for motion monitoring. *Nat. Commun.* **11**, 3530 (2020). 32669576 10.1038/s41467-020-17301-6 PMC7363829

[29] Du, K. et al. Electronic textiles for energy, sensing, and communication. *iScience* **25**, 104174 (2022). 35479405 10.1016/j.isci.2022.104174 PMC9035708

[30] Ray, T. R. et al. Bio-integrated wearable systems: a comprehensive review. *Chem. Rev.* **119**, 5461–5533 (2019). 30689360 10.1021/acs.chemrev.8b00573

[31] Lee, J. et al. Stretchable and suturable fibre sensors for wireless monitoring of connective tissue strain. *Nat. Electron.* **4**, 291–301 (2021).

[32] Fadeel, B. et al. Safety assessment of graphene-based materials: focus on human health and the environment. *ACS Nano* **12**, 10582–10620 (2018). 30387986 10.1021/acsnano.8b04758

[33] Park, J. et al. Frequency-selective acoustic and haptic smart skin for dual-mode dynamic/static human-machine interface. *Sci. Adv.* **8**, eabj9220 (2022). 35333568 10.1126/sciadv.abj9220 PMC8956263

[34] Park, K. et al. A biomimetic elastomeric robot skin using electrical impedance and acoustic tomography for tactile sensing. *Sci. Robot.* **7**, eabm7187 (2022). 35675452 10.1126/scirobotics.abm7187

[35] Liu, Y. et al. Epidermal mechano-acoustic sensing electronics for cardiovascular diagnostics and human-machine interfaces. *Sci. Adv.* **2**, e1601185 (2016). 28138529 10.1126/sciadv.1601185 PMC5262452

[36] Nayeem, M. O. G. et al. All-nanofiber–based, ultrasensitive, gas-permeable mechanoacoustic sensors for continuous long-term heart monitoring. *Proc. Natl Acad. Sci. USA* **117**, 7063–7070 (2020). 32188781 10.1073/pnas.1920911117 PMC7132136

[37] Ozcelik, A. et al. Acoustic tweezers for the life sciences. *Nat. Methods* **15**, 1021–1028 (2018). 30478321 10.1038/s41592-018-0222-9 PMC6314293

[38] Li, P. et al. Acoustic separation of circulating tumor cells. *Proc. Natl Acad. Sci. USA* **112**, 4970–4975 (2015). 25848039 10.1073/pnas.1504484112 PMC4413297

[39] Ahmed, D. et al. Selectively manipulable acoustic-powered microswimmers. *Sci. Rep.* **5**, 9744 (2015). 25993314 10.1038/srep09744 PMC4438614

[40] Han, J., Zhang, L. & Leus, G. Partial FFT demodulation for MIMO-OFDM over time-varying underwater acoustic channels. In *Proc. IEEE Signal Processing Letters* Vol. 23 282–286 (IEEE, 2016).

[41] Wang, Y., Hu, R., Chen, Y. & Huang, S. H. Adaptive noise cancelling for an AUV-mounted passive inverted USBL array. *Ocean Eng.* **288**, 115998 (2023).

[42] Grami, A. in *Introduction to Digital Communications* (ed. Grami, A.) 457–491 (Academic, 2016).

[43] Daniel, R. & Tony, V.-B. in *Elastic Waves in Solids 1* (eds Favennec, P.-N. & de Fornel, F.) 179–246 (Wiley, 2022).

[44] Kim, J. M., Marashi, C., Wee, J. & Peters, K. Acoustic wave coupling between optical fibers of different geometries. *Appl. Opt.* **60**, 11042–11049 (2021). 35201092 10.1364/AO.441494

[45] Nienwenhui, J. H., Neumann, J. J., Greve, D. W. & Oppenheim, I. J. Generation and detection of guided waves using PZT wafer transducers. In *Proc. IEEE Transactions on Ultrasonics, Ferroelectrics, and Frequency Control* Vol. 52 2103–2111 (IEEE, 2005). 10.1109/tuffc.2005.1561681 16422424

[46] Xu, C., Solomon, S. A. & Gao, W. Artificial intelligence-powered electronic skin. *Nat. Mach. Intell.* **5**, 1344–1355 (2023). 38370145 10.1038/s42256-023-00760-z PMC10868719

[47] Luo, Y. et al. Learning human–environment interactions using conformal tactile textiles. *Nat. Electron.* **4**, 193–201 (2021).

[48] Li, D. et al. Touch IoT enabled by wireless self-sensing and haptic-reproducing electronic skin. *Sci. Adv.* **8**, eade2450 (2022). 36563155 10.1126/sciadv.ade2450 PMC9788763

[49] Cai, H. et al. Brain organoid reservoir computing for artificial intelligence. *Nat. Electron.* **6**, 1032–1039 (2023).

[50] Safari, A. & Akdoğan, E. K. *Piezoelectric and Acoustic Materials for Transducer Applications* (Springer, 2008).

[51] Jin, H. et al. Review on piezoelectric actuators based on high-performance piezoelectric materials. In *Proc. IEEE Transactions on Ultrasonics, Ferroelectrics, and Frequency Control* Vol. 69 3057–3069 (IEEE, 2022). 10.1109/TUFFC.2022.3175853 35580107

[52] Wang, H. W., Zhou, H. W., Gui, L. L., Ji, H. W. & Zhang, X. C. Analysis of effect of fiber orientation on Young’s modulus for unidirectional fiber reinforced composites. *Compos. Part B* **56**, 733–739 (2014).

[53] Giurgiutiu, V. in *Structural Health Monitoring with Piezoelectric Wafer Active Sensors* 2nd edn (ed. Giurgiutiu, V.) 199–292 (Academic, 2014).

