# Supplementary material for: A smart acoustic textile for health monitoring
Source: Nat Electron. 2025 May 19;8(6):485–95. doi: 10.1038/s41928-025-01386-2 (PMC12202489; doi:10.1038/s41928-025-01386-2)
Supplement: Supplementary file 1 — Supplementary Notes 1–6, Figs. 1–18 and References. [file 41928_2025_1386_MOESM1_ESM.pdf]

nature electronics

Supplementary information

https://doi.org/10.1038/s41928-025-01386-2

A smart acoustic textile for health monitoring

In the format provided by the authors and unedited

TABLE OF CONTENTS

Supplementary Notes (Page 2-4)

Suppl. Note 1. Selection of working frequency of SonoTextiles

Suppl. Note 2. Crosstalk between channels of the tactile sensing array

Suppl. Note 3. Sensitivity of SonoTextiles under various weaving conditions

Suppl. Note 4. Impact of textile materials and thickness on SonoTextiles

Suppl. Note 5. Effective wave propagation distance in SonoTextiles

Suppl. Note 6. Response time of SonoTextiles

Supplementary Figures (Page 5-22)

Suppl. Fig. 1 | Single-input single-output SonoTextiles.

Suppl. Fig. 2 | Experimental setup for basic evaluations of the SISO SonoTextiles.

Suppl. Fig. 3 | Impedance characteristics of the PZT transducers.

Suppl. Fig. 4 | Characterization of SISO SonoTextiles in different weaving conditions.

Suppl. Fig. 5 | Experimental setup for the external pressing force perception by SISO SonoTextiles.

Suppl. Fig. 6 | Simulation analysis on a four-channel multi-input single-output system.

Suppl. Fig. 7 | Comparison of system complexity between the fibre array and traditional dot matrix array.

Suppl. Fig. 8 | Schematic diagrams of the SonoGloves.

Suppl. Fig. 9 | Schematic diagrams of the respiratory monitoring by SonoTextiles.

Suppl. Fig. 10 | Evaluation of the washability and thermal stability of SonoTextiles.

Suppl. Fig. 11 | Dealing with the crosstalk between channels.

Suppl. Fig. 12 | Sensitivity comparison of SonoTextiles to applied pressing forces under various weaving conditions.

Suppl. Fig. 13 | Evaluation of the stability and durability of SonoTextiles.

Suppl. Fig. 14 | Feasibility and performance comparison of SonoTextiles with textile substrates of different materials.

Suppl. Fig. 15 | Experimental analysis of the impact of textile substrate thickness on wave propagation in SonoTextiles.

Suppl. Fig. 16 | Evaluation of wave travel distances in SonoTextiles with and without a textile substrate, assessed through the Rx amplitude.

Suppl. Fig. 17 | Observation of wave propagation delay in SISO SonoTextiles.

Suppl. Fig. 18 | Temperature variation during continuous operation of two types of PZTs.

Supplementary References (Page 23)

Supplementary Notes

Supplementary Note 1. Selection of working frequency of SonoTextiles

In order to improve the output efficiency of the transducer, we conducted impedance analysis on the PZT transducer used by SonoTextiles, as illustrated in **Supplementary Fig. 3**. The 27-mm-diameter transducer (7BB-27-4L0, Murata Electronics) was subjected to a sweep frequency test from 30 kHz to 200 kHz. The test results showed that the phase angle of the 27-mm-diameter transducer was 0 at 101 kHz, and the impedance was small, indicating that the mechanical conversion efficiency near this frequency was the highest. Therefore, we selected the frequency near this frequency as the excitation frequency, that is, 98~104 kHz. And the impedance analysis in **Supplementary Fig. 3** showed that when the frequency was 110 kHz, the impedance was the largest, proving that the transducer was heating the most seriously, so this frequency should be avoided when selecting the excitation frequency.

For the 10-mm-diameter transducer (SMD10T2R111WL, STEMINC), we conducted a sweep frequency analysis from 50 kHz to 300 kHz. As the phase angle of the transducer was gradually increased, multiple zero crossings were observed within the frequency range of 174 to 184 kHz. The transducer impedance in this interval was low, making it an optimal choice for the excitation frequency. Although there is also a zero-crossing point near 200 kHz, the high impedance at this frequency can lead to excessive heating and potential damage to the transducer. Therefore, this frequency range should be avoided.

Supplementary Note 2. Crosstalk between channels of the tactile sensing array

Crosstalk refers to the interference caused by the coupling of signals between different channels[1]. If not properly prevented or managed, crosstalk can lead to erroneous outputs or reduced resolution in array sensing[2,3]. As we indicated in the main text (**Fig. 3**), we simultaneously use the four frequencies along the weft and warp directions of the 4×4 fibre-array tactile sensing system. The same frequency on the warp and weft threads may interfere with the frequency-division-multiple-access (FDMA) addressing in a certain direction due to crosstalk phenomena. Therefore, we have implemented effective preventive methods during the design process to avoid crosstalk issues. As discussed in **Supplementary Fig. 11**, at each intersection point of the fibre array, the warp and weft threads pass through the textile substrate from different sides, with the warp passing through from the front side and the weft from the back side. This approach ensures that there will be no direct contact between the warp and weft threads, effectively mitigating inter-channel crosstalk. The spectra provided as an example in **Supplementary Fig. 11c** effectively demonstrates that the fibre-array tactile sensing system has not been affected by crosstalk issues.

Supplementary Note 3. Sensitivity of SonoTextiles under various weaving conditions

Sensitivity is a crucial metric for SonoTextiles, and to address this, we conducted experiments comparing the sensitivity of SonoTextiles to applied pressing forces under various conditions.

Specifically, we tested the system under four conditions: without a textile substrate and with textile substrates featuring stitch densities of 6, 12, and 18 stitches per 5 cm. The results are presented in **Supplementary Fig. 12**, which plots the reception amplitude of the R_x transducer versus pressing force for these four conditions in the SISO SonoTextiles configuration.

As shown in **Supplementary Fig. 12**, SonoTextiles exhibit a strong response to pressing forces across all conditions, with higher sensitivity observed during the initial phase of increasing pressing forces. This demonstrates the reliability and robustness of SonoTextiles' sensing capabilities. Additionally, we compared the sensitivity by evaluating the energy loss of the R_x amplitude (relative changes in dB) as the pressing force increased from 0 to 7.8 N. The results revealed energy losses of 24.8 dB, 17.3 dB, 15.5 dB, and 8.1 dB at a pressing force of 7.8 N for the four conditions, respectively. These findings suggest that the sensitivity of SonoTextiles is influenced by the weaving conditions, with higher stitch densities potentially leading to a decrease in sensitivity.

Supplementary Note 4. Impact of textile materials and thickness on SonoTextiles

The material and thickness of the textile substrate are critical factors influencing the performance of SonoTextiles. In our study, we utilized a widely available fabric composed of a two-thirds cotton and one-third polyester blend, as shown in **Fig. 2** and **Fig. 3**. This fabric was selected with a priority on breathability, ensuring both comfort and functionality. Furthermore, SonoTextiles has demonstrated robustness and adaptability across various textile substrates, indicating its versatility for diverse applications.

To investigate the impact of textile substrate material on SonoTextiles, we conducted a comparative experiment using six different fabrics: wool, silk, linen, cotton, nylon, and polyester, as shown in **Supplementary Fig. 14 (a-f)**. The results, presented in **Supplementary Fig. 14g**, show that, despite some variations in the R_x reception amplitude, SonoTextiles exhibited similar input-output characteristics across the different fabric materials. Additionally, as illustrated in **Supplementary Fig. 14h**, SonoTextiles demonstrated an effective response to periodic pressing forces regardless of the material.

We also explored the effect of textile substrate thickness on wave propagation in SonoTextiles. **Supplementary Fig. 15 (a-c)** presents input-output characterization experiments conducted using linen substrates of three different thicknesses, with identical material and weaving. As shown in **Supplementary Fig. 15d**, under the same experimental conditions (101 kHz, 12 stitches per 5 cm), the thickness of the textile substrate significantly impacted the R_x signal amplitude. This observation is consistent with the expectation that a thicker textile substrate absorbs more acoustic wave energy due to the increased fibre contact area, resulting in greater energy dissipation during propagation.

Supplementary Note 5. Effective wave propagation distance in SonoTextiles

The effective wave travel distance is a crucial factor in determining the performance of SonoTextiles, and understanding its limits is essential for optimizing its functionality. To explore this issue, we conducted experiments to assess the wave travel distance along the fibre,

with and without the textile substrate, as presented in **Supplementary Fig. 16**. Firstly, we examined the R_x amplitude for a SISO SonoTextile without a textile substrate, as shown in **Supplementary Fig. 16a**. As the fibre length increases from 100 cm to 300 cm, the Rx amplitude decreases due to propagation loss, but the Rx amplitude remains as high as 75 mV for fibre lengths up to 300 cm. This result highlights the capability of acoustic waves to propagate over long distances along the fibre in the absence of the textile substrate. Secondly, we investigated the effect of different weaving conditions on R_x amplitude for SonoTextiles with a textile substrate, as shown in **Supplementary Fig. 16b**. Under the same stitch density, the R_x amplitude shows noticeable attenuation as the fibre length increases. However, the attenuation is less pronounced with 6 stitches per 5 cm compared to 12 stitches per 5 cm, allowing for effective propagation distances of approximately 35 cm and 20 cm, respectively.

These results emphasize that the effective propagation distance in SonoTextiles can be significantly influenced by factors such as textile substrate and weaving conditions. Furthermore, as demonstrated by the propagation distance exceeding 300 cm without the textile substrate, there is significant potential for optimizing the effective range of SonoTextiles through advancements in PZT transducers, microfiber materials, textiles, and fabrications. Such optimizations could further expand their applications in wearable technologies.

Supplementary Note 6. Response time of SonoTextiles

The response time of SonoTextiles refers to the time taken for the system to detect and react to applied stimuli. Through both analysis and experimental verification, we found that the response time of SonoTextiles is at the microsecond (μs) level, indicating an exceptionally fast reaction time.

In the single-input-single-output (SISO) system, the acoustic wave is emitted by the T_x PZT, propagates through the fibre waveguide, and reaches the R_x PZT. This process is rapid, as the speed of acoustic waves in glass fibres is typically several kilometers per second[4]. Additionally, the electro-acoustic conversion by the PZT occurs in microseconds[5], further contributing to the system's quick response. To quantify this, we conducted an experiment using a pulse signal in the SISO system to measure the signal propagation delay. The system was configured with two oscilloscope channels connected to the T_x PZT and R_x PZT, with a fibre length of 15 cm. As shown in **Supplementary Fig. 17**, a delay of 50 μs was observed in the signal reaching the R_x PZT, which aligns with our expectations. The response of SonoTextiles arises from the disruption of acoustic wave propagation by external stimuli. Therefore, the response time of the system should also in the microsecond level.

Supplementary Figures

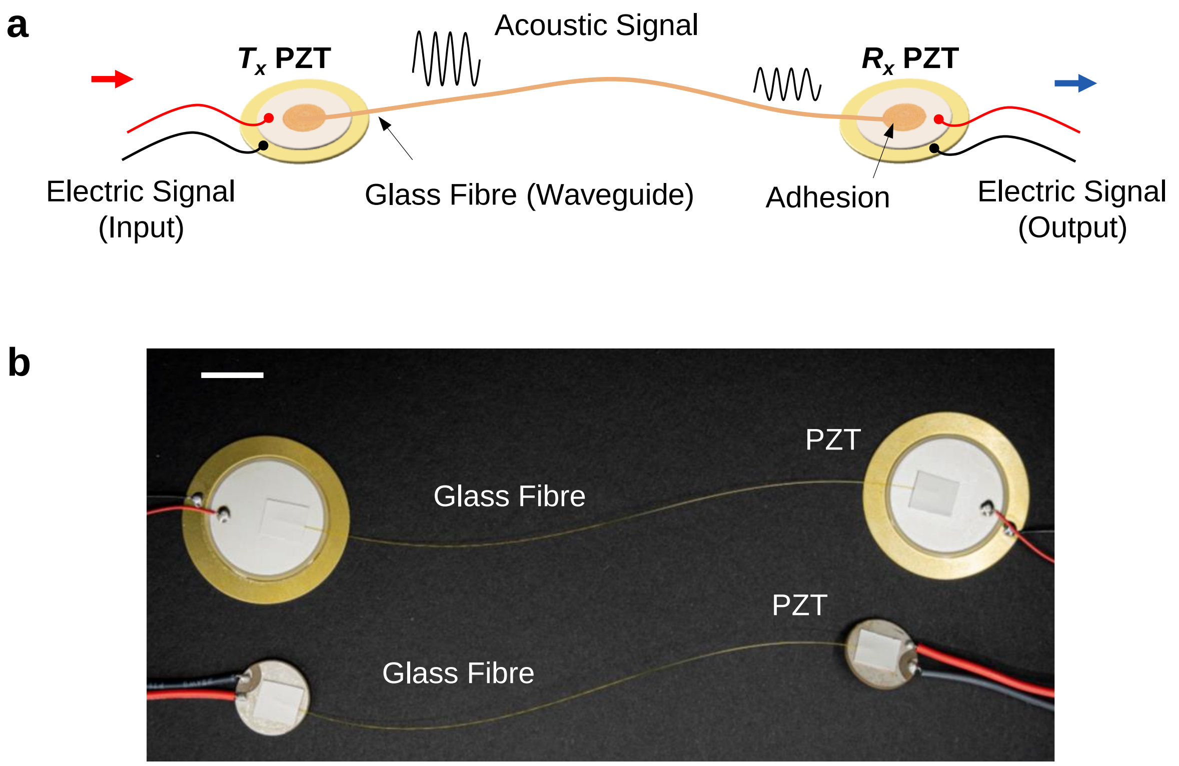

Supplementary Fig. 1 | Single-input single-output SonoTextiles. (a) Schematic of the basic single-input- single-output system without textile substrate. The T_x PZT performs electroacoustic conversion based on the input electric signal, outputting acoustic waves. The acoustic signal propagates along the silica fibre, which acts as the acoustic waveguide, to the R_x PZT. There is natural attenuation of the acoustic waves during propagation, and if external stimuli are applied, the attenuation will increase accordingly. The R_x PZT receives the incoming acoustic waves and performs the acoustoelectric conversion, outputting electrical signals for subsequent measurements. (b) Image of two single-input single-output systems before being woven into textile substrates, where two different models of PZT are employed. The smaller model of PZT is more suitable for wearable applications. Scale bar, 10 mm.

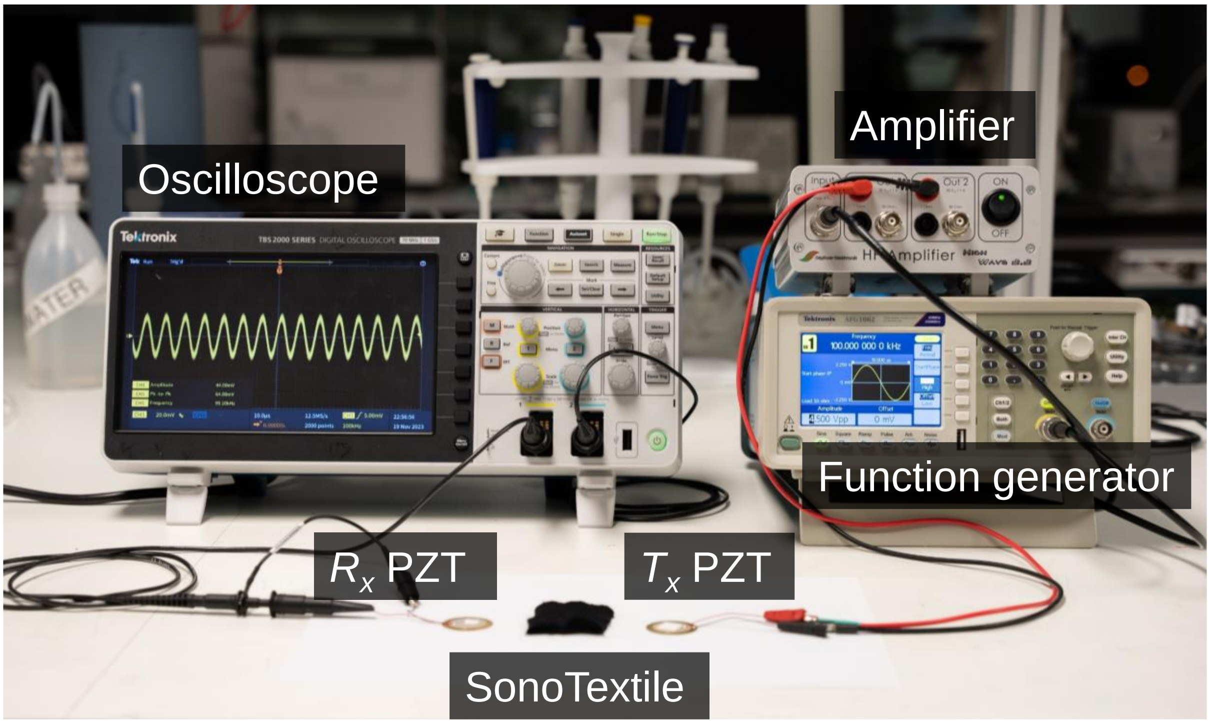

Supplementary Fig. 2 | Experimental setup for basic evaluations of the single-input single-output SonoTextiles. The function generator outputs electrical signals, which are amplified by the power amplifier before being connected to the T_x PZT. The acoustic signal propagates through the microfiber waveguide and reaches the R_x PZT. The oscilloscope measures and records the electrical signals obtained from the R_x PZT by acoustoelectric conversion.

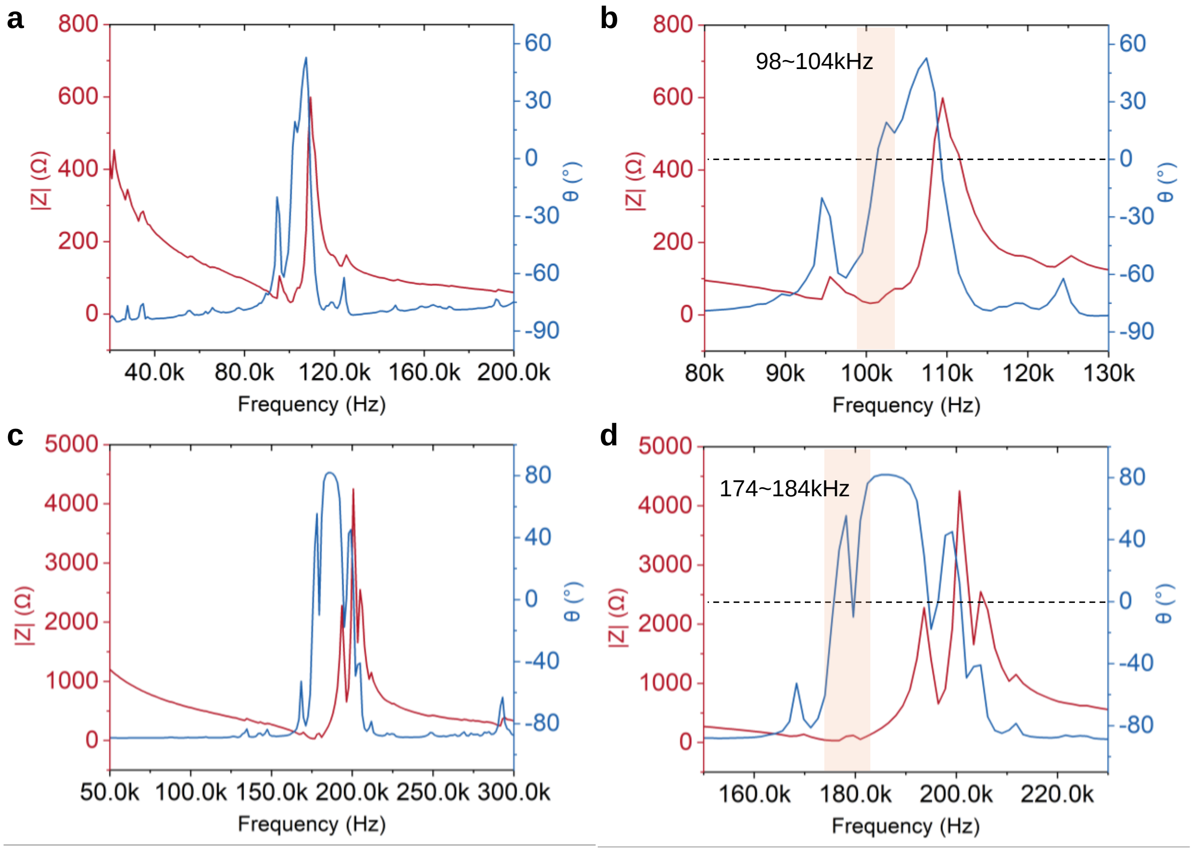

Supplementary Fig. 3 | Impedance characteristics of the PZT transducers. (a) Impedance test result of the 27-mm-diameter transducer (7BB-27-4L0, Murata Electronics). (b) The zoomed-in view of (a). The frequency band from 98 kHz to 104 kHz is chosen for the characterization evaluation of the SISO system and fibre-array tactile sensing experiments. (c) Impedance test result of the 10-mm-diameter transducer (SMD10T2R111WL, STEMINC). (d) The zoomed-in view of (c). The frequency band from 174 kHz to 184 kHz is chosen for the SonoGloves and physiological monitoring applications.

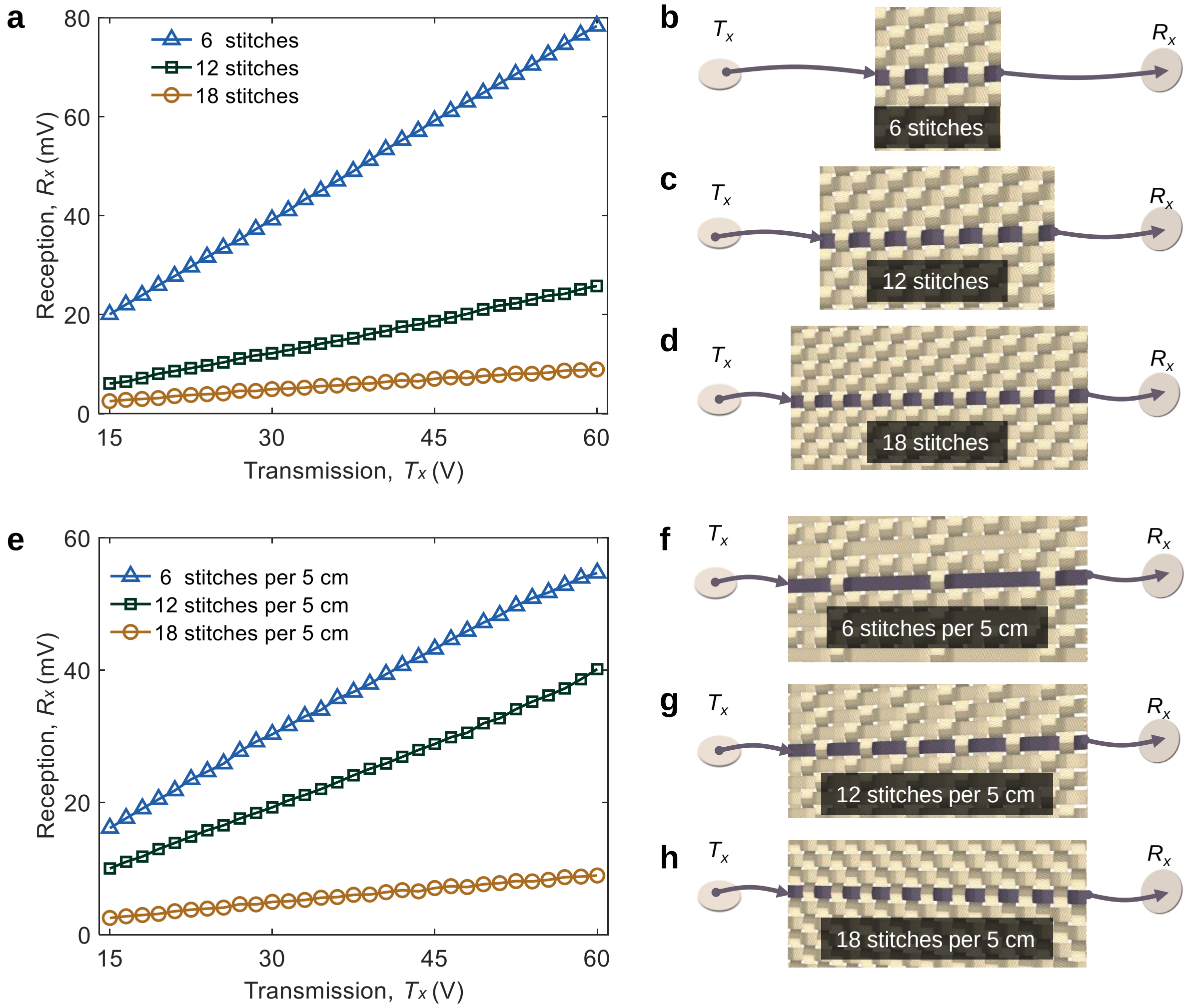

Supplementary Fig. 4 | Characterization of the single-input single-output SonoTextiles in different weaving conditions. (a) Plot of R_x reception versus T_x transmission peak-to-peak amplitude, where the fibre is woven into the textile substrate in the same stitch density with different stitch numbers. The T_x and R_x signal amplitudes are directly proportional in the three cases. The larger the stitch number, the more significant the attenuation of the R_x signal amplitude. (b, c, d) Schematic diagrams of the above three cases with different stitch numbers (6 stitches, 12 stitches, 18 stitches), where the stitch density is 18 stitches per 5 cm. (e) Plot of R_x reception versus T_x transmission peak-to-peak amplitude, where the fibre is woven in different stitch densities in the same textile substrate. The greater the stitch density, the more significant the attenuation of the R_x signal amplitude. (f, g, h) Schematic diagrams of the three cases with different stitch densities (6 stitches per 5 cm, 12 stitches per 5 cm, 18 stitches per 5 cm).

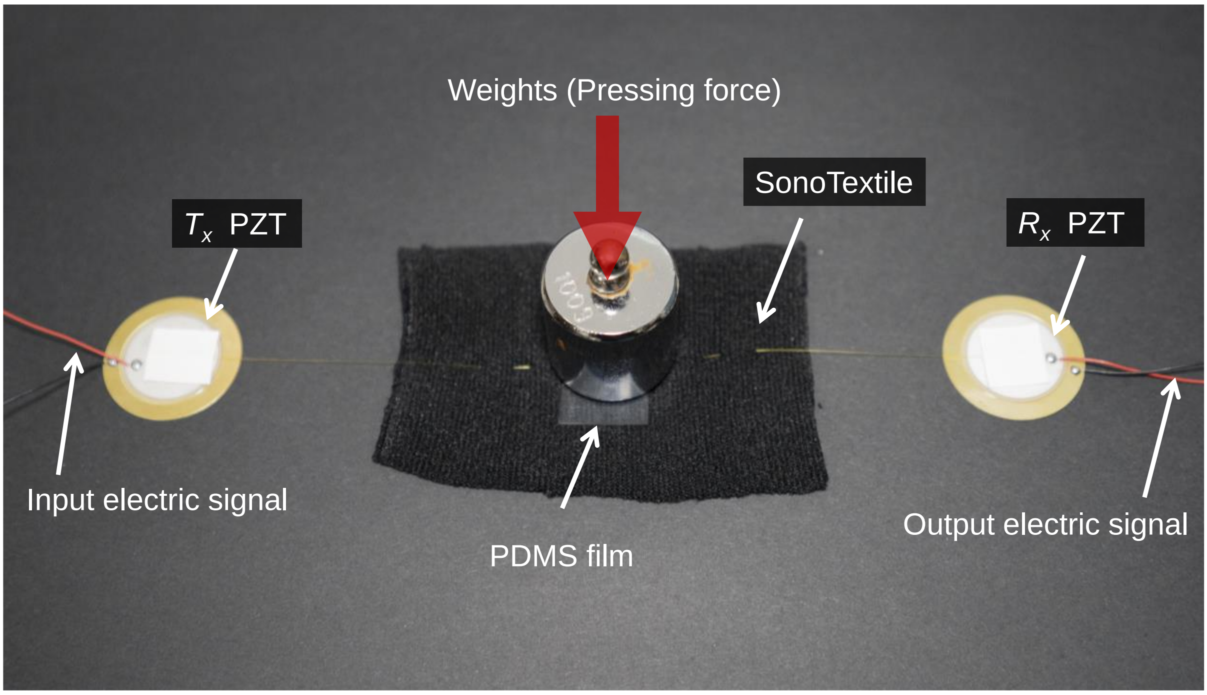

Supplementary Fig. 5 | Experimental setup for the external pressing force perception by the single-input single-output SonoTextiles. We used weights as a quantitative source of external pressing force and. The pressing force was applied on the textile substrate where the glass microfiber was woven. A polydimethylsiloxane (PDMS) film was placed between the SonoTextiles and the weights to simulate human skin and ensure consistent contact areas.

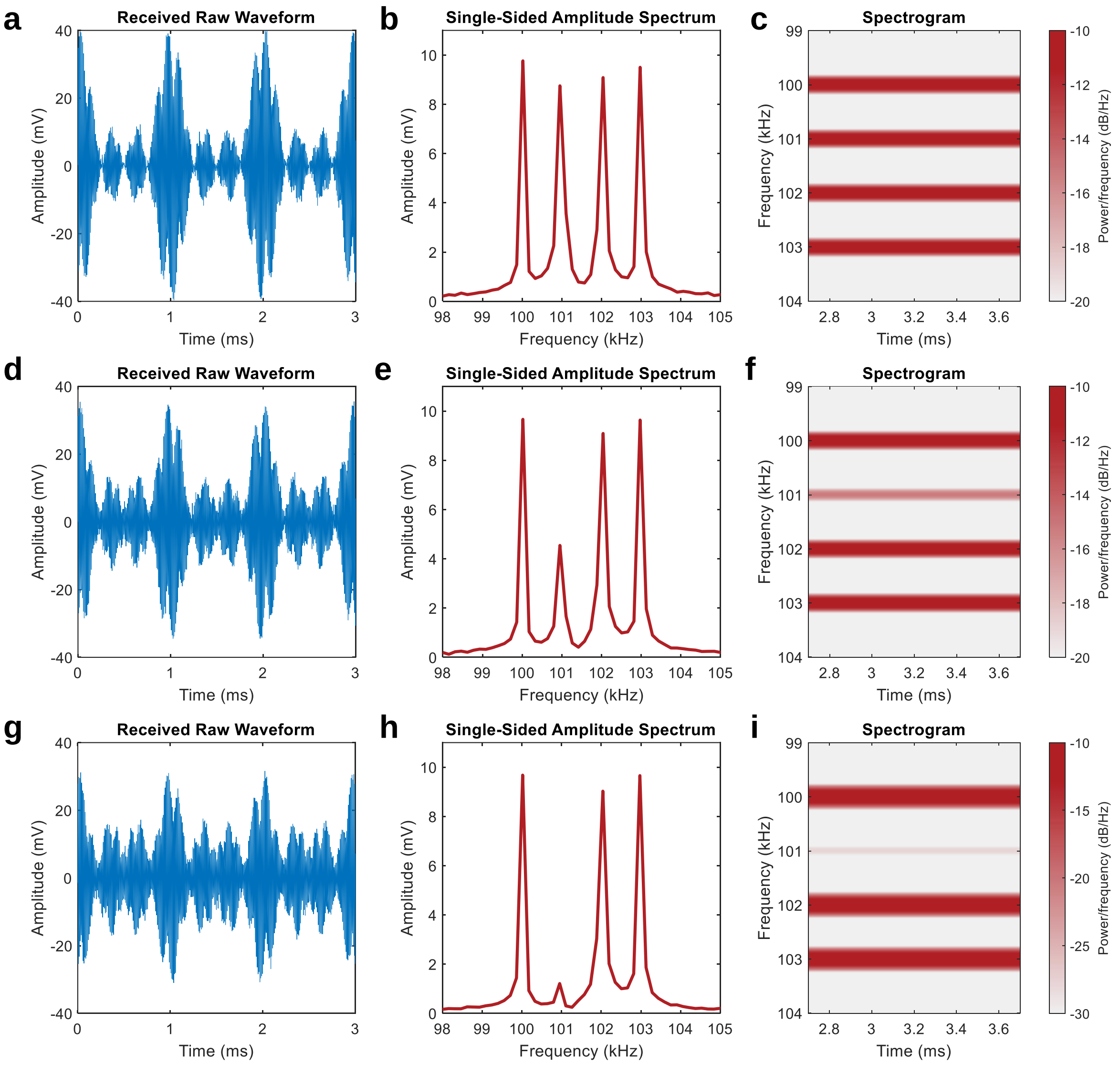

Supplementary Fig. 6 | Simulation analysis on a four-channel multi-input single-output system. (a) Received superimposed signals from the four channels that are assigned frequencies of 100 kHz, 101 kHz, 102 kHz, and 103 kHz respectively, in the natural state without external stimuli. (b) The single-sided amplitude spectrum obtained by fast Fourier transform. Four obvious peaks appear at the corresponding four frequencies. The slight inconsistency between the peaks is because we added noise to the signals (the signal-to-noise ratio was 1). (c) The time-frequency spectrogram obtained by short-time Fourier transform, presented in terms of power. The obvious and continuous bright bands at the four frequencies indicate the natural state of the system. (d, e, f) The received raw waveform, single-sided spectrum, and the time-frequency spectrogram when the second channel (101 kHz) experienced a 3 dB amplitude attenuation (6 dB power attenuation) due to external stimuli. The peak of 101 kHz in the spectrum and the band brightness around 101 kHz in the spectrogram were obviously reduced. (g, h, i) The simulated results when the second channel experienced a 9 dB amplitude attenuation (18 dB power attenuation). The second channel (101 kHz) was almost blocked.

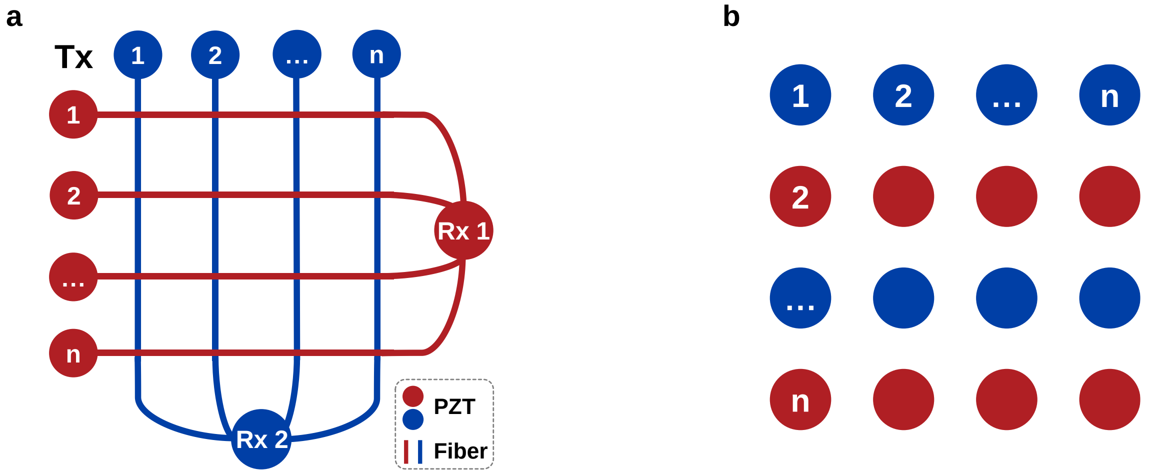

Supplementary Fig. 7 | Comparison of system complexity between the fibre array and traditional dot matrix array. (a) Illustration of the proposed fibre-array tactile sensing interface. The fibre array owns n T_x transducers and one R_x transducer in the weft and warp directions respectively, forming a sensing array with $n \times n$ touch points. (b) Illustration of a traditional dot matrix sensing array. The array has n rows, each with n transducers (or other sensors), providing $n \times n$ touch points. SonoTextile needs $2 \times (n+1)$ sensors, while the traditional dot matrix approach needs $n \times n$ sensors.

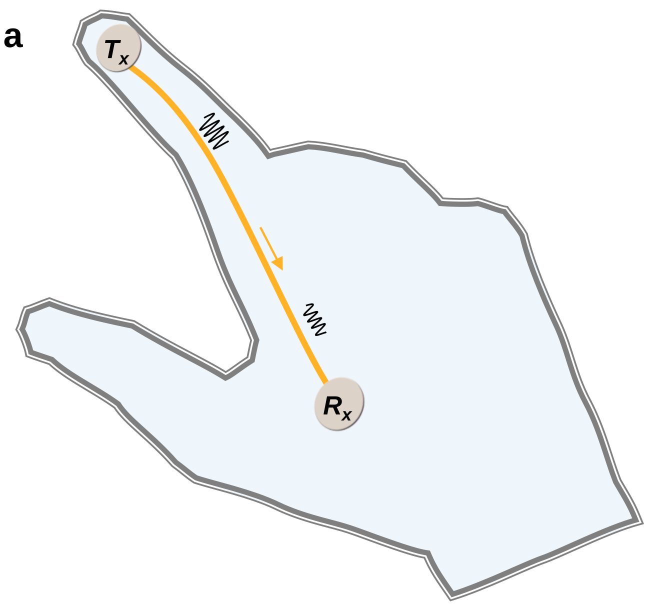

Supplementary Fig. 8 | Schematic diagrams of the SonoGloves. (a) Illustration of SonoGloves for finger bending angle perception using a single-input single-output SonoTextile. (b) Illustration of SonoGloves for gesture recognition using a multi-input single-output SonoTextile. Silica fibres are woven into the glove textile substrate, running along the five fingers. The five T_x PZT elements are allocated different frequencies as indicated in the figure from f_1 to f_5. The signal processing for the gesture recognition is based on the FDMA in the frequency domain.

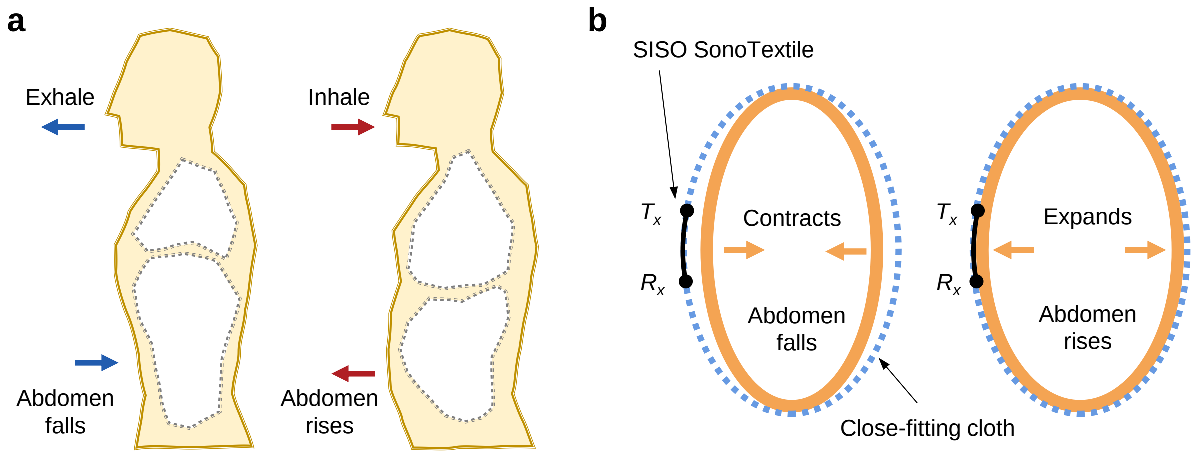

Supplementary Fig. 9 | Schematic diagrams of the respiratory monitoring by SonoTextiles. (a) A basic illustration of human abdominal breathing. The rhythmic rise and fall of the belly during the inhalation and exhalation can be utilized for monitoring respiratory rate. (b) A top-down schematic of the SonoTextiles system used for respiratory monitoring. A single-input single-output system is woven into the abdominal area of the close-fitting cloth. When the abdomen contracts (abdomen falls), the acoustic wave propagation between the T_x and R_x PZT elements is undisturbed. But when the abdomen expands (abdomen rises), the abdominal skin will come into contact with the SonoTextiles system. This contact force is a kind of external pressure which would lead to a significant wave energy loss in the R_x end.

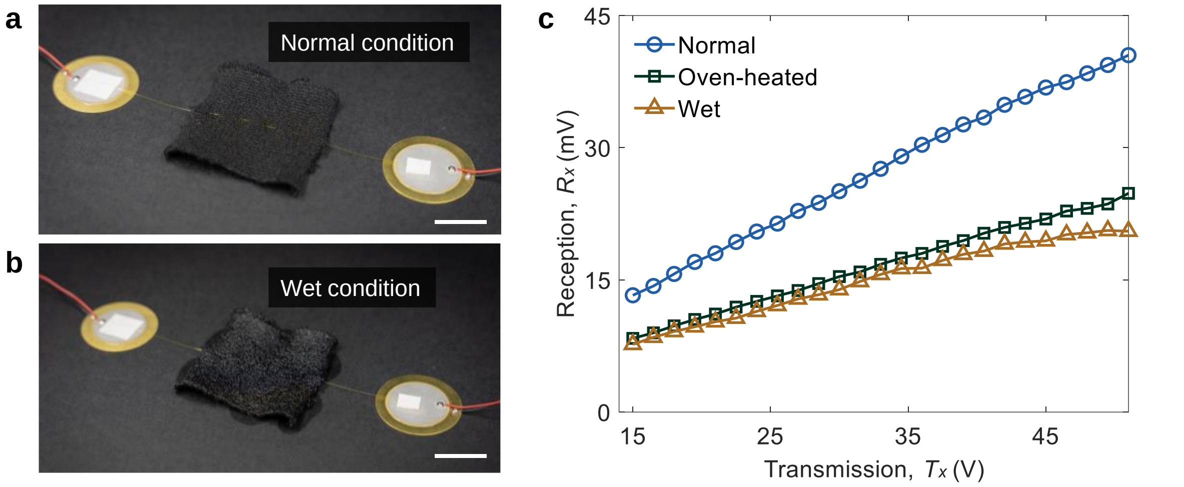

Supplementary Fig. 10 | Evaluation of the washability and thermal stability of SonoTextiles. (a) The single-input single-output system in the normal condition. The textile substrate was dry at the room temperature of about 20°C. Scale bar, 10mm. (b) The single-input single-output system in the wet condition. The textile substrate was soaked with water to evaluate its washability. Scale bar, 10mm. (c) Plot of R_x reception versus T_x transmission peak-to-peak amplitude of a single-input single-output system under three different conditions. In the heated condition, the system was heated in an oven at 86° for 20 minutes before the evaluation. The heated system still exhibits great acoustic input and output performance. However, the R_x signal amplitude is smaller compared to the normal state, possibly due to heating reducing the adhesion strength between the fibre and the PZT, thereby increasing propagation loss. Similarly, in the wet condition, the R_x signal amplitude is smaller compared to the normal state. We believe this is mainly because the wet textile substrate makes more complete contact with the fibre, leading to more significant propagation loss.

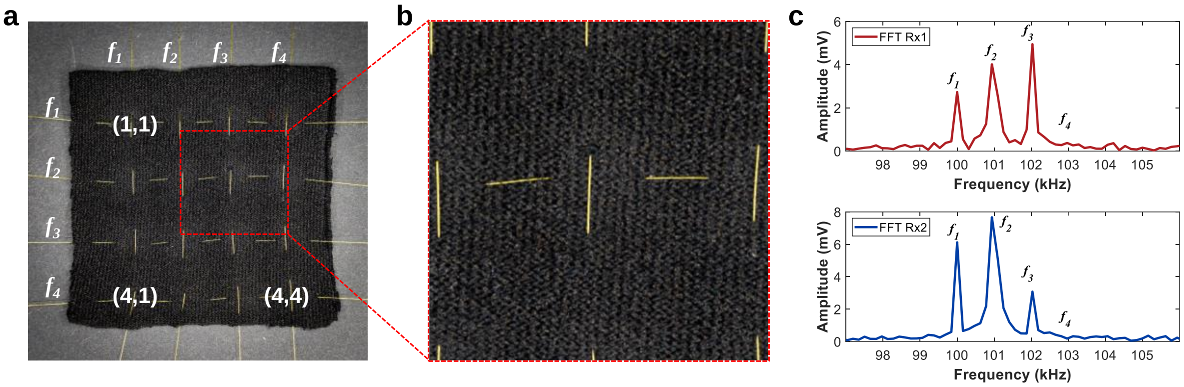

Supplementary Fig. 11 | Dealing with the crosstalk between channels. (a) Image of the 4×4 acoustic fibre-array tactile sensing interface. Consistent with the definitions in the main text, the coordinates of the upper left and lower right touch points are defined as (1, 1) and (4, 4) respectively. (b) Zoomed-in view of the array near the coordinate (2, 3). The warp and weft threads pass through the textile substrate from the front and back sides at each touch point, respectively. Under this strategy, there will be no direct contact between the warp and weft threads, thereby preventing inter-channel crosstalk. (c) Single-sided amplitude spectra obtained though FFT when the coordinate (4, 4) is pressed. If there is crosstalk between the warp and weft channels, a signal with the frequency of 103 kHz (f_4) from the warp would appear in the spectrum of the weft receiver R_x 1. Conversely, the same would occur for R_x 2. However, the absence of a significant 103 kHz signal amplitude in the spectra of both R_x 1 and R_x 2 indicates that the system is not affected by crosstalk.

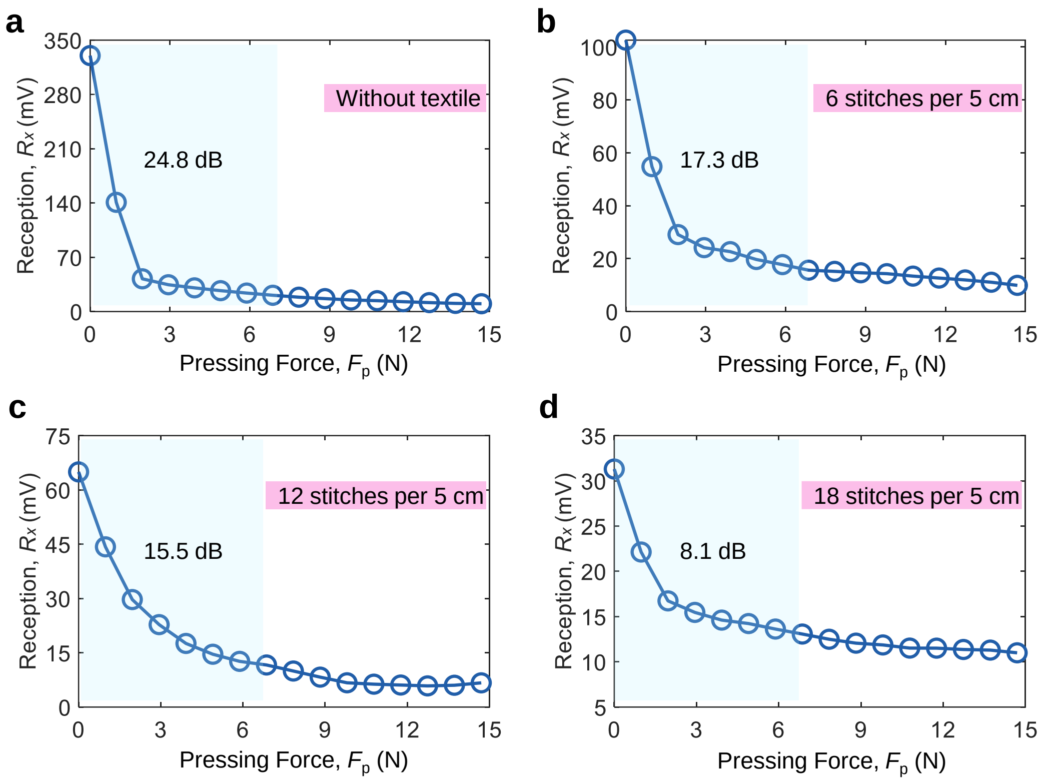

Supplementary Fig. 12 | Sensitivity comparison of SonoTextiles to applied pressing forces under various weaving conditions. (a) R_x reception amplitude versus pressing force for a SISO SonoTextile without the textile substrate. (b, c, d) R_x reception amplitude versus pressing force for a SISO SonoTextile with textile substrates featuring stitch densities of 6, 12, and 18 stitches per 5 cm. Under these four conditions, SonoTextiles exhibited energy losses of 24.8 dB, 17.3 dB, 15.5 dB, and 8.1 dB at a pressing force of 7.8 N, respectively. The sensitivity of SonoTextiles is influenced by the weaving condition, with higher stitch densities potentially resulting in a decrease in sensitivity.

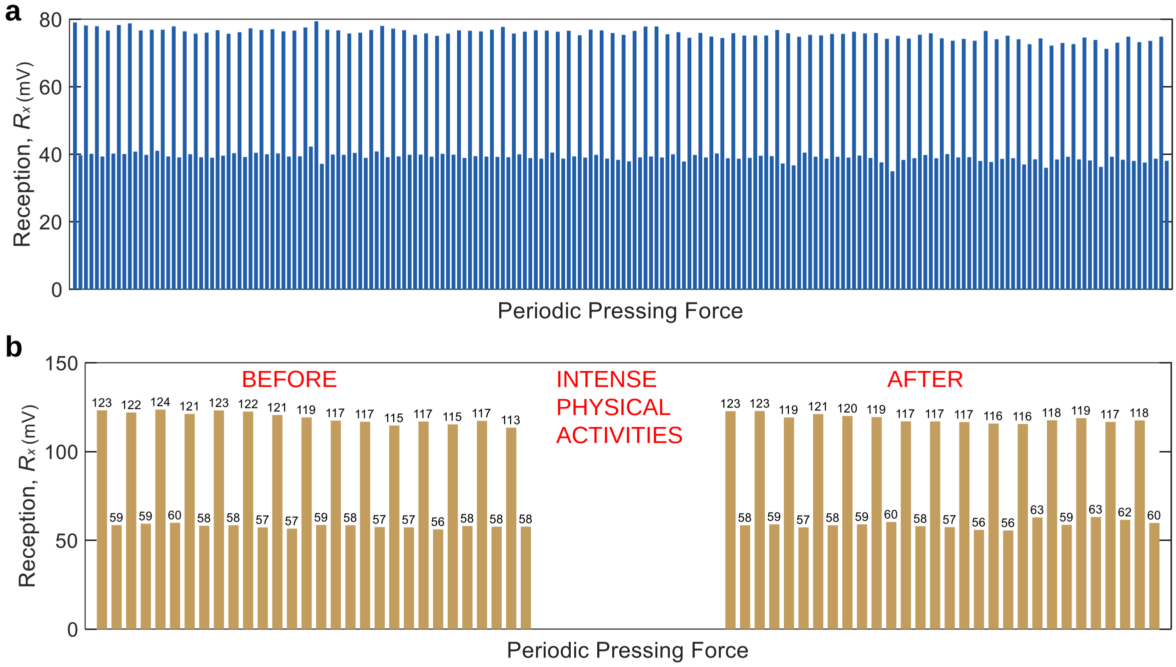

Supplementary Fig. 13 | Evaluation of the stability and durability of SonoTextiles. (a) Stability and performance consistency of the SISO SonoTextiles under repeated external stimuli. A 100 g weight was applied to deliver consistent pressure stimuli, and 100 repetitions were performed. The R_x amplitude was measured before and after each stimulus. The results show that SonoTextiles maintained stable and consistent input-output performance, demonstrating its robustness and stability. (b) Durability of SonoTextiles under intense physical stress. SonoTextiles was subjected to repeated bending, rubbing, and pounding. We applied 15 repeated pressing stimuli using a 100 g weight, both before and after the intense physical activities. SonoTextiles remained undamaged and exhibited stable, consistent response performance, confirming the durability and reliability of the system under rigorous conditions.

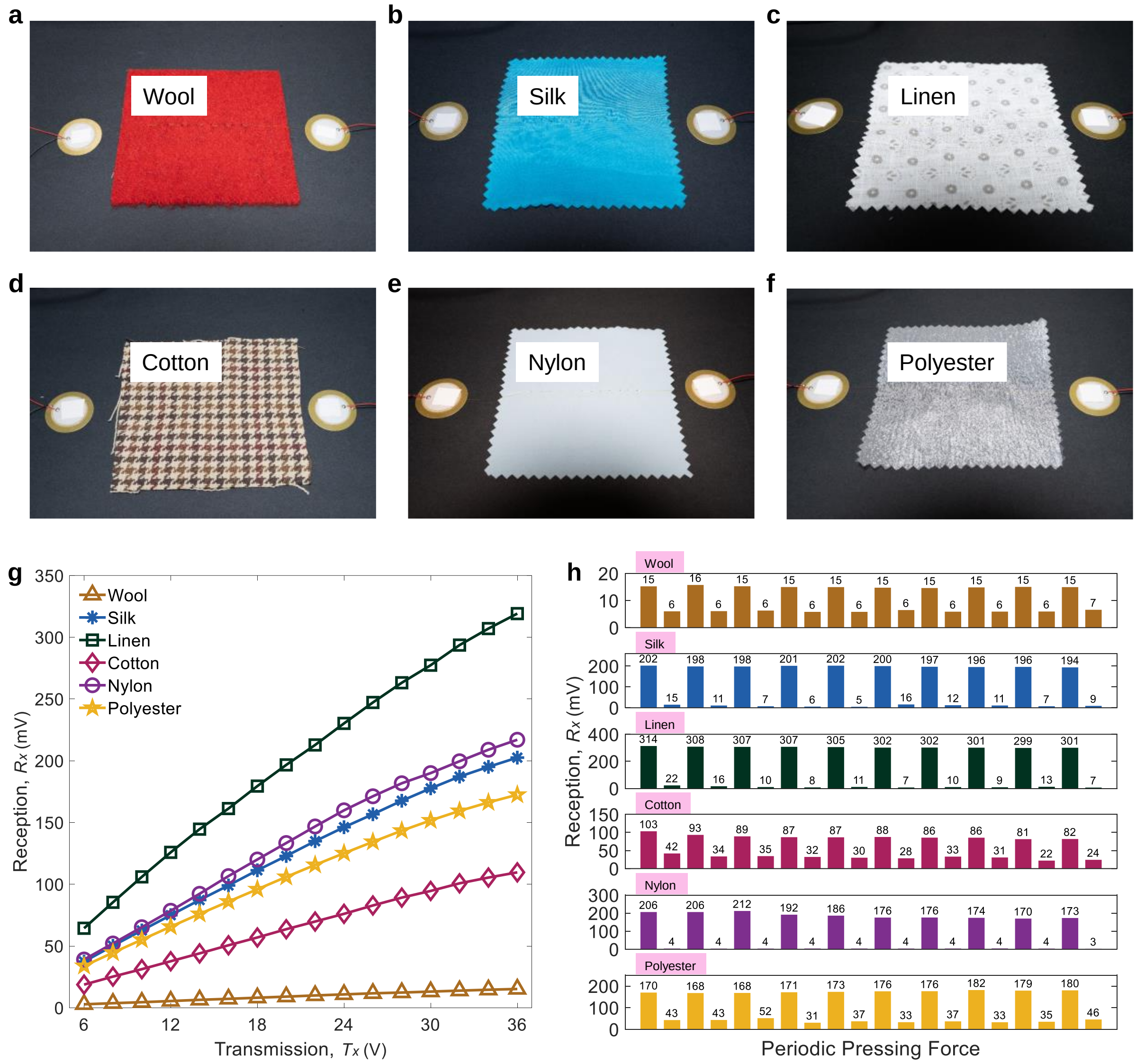

Supplementary Fig. 14 | Feasibility and performance comparison of SonoTextiles with textile substrates of different materials. (a-f) Images of SISO SonoTextiles using textile substrates made from six different materials: wool, silk, linen, cotton, nylon, and polyester. (g) Input-output characteristics of SonoTextiles across different fabric materials. Although there are some variations in the R_x reception amplitude, SonoTextiles with different textile substrates exhibit stable transmission and reception characteristics. (h) Responses of SonoTextiles across different fabric materials to periodic pressing forces. A 100 g weight was used to provide a stable, repetitive pressing force. It can be seen that SonoTextiles, with different textile substrates, exhibit effective and stable responses to external stimuli.

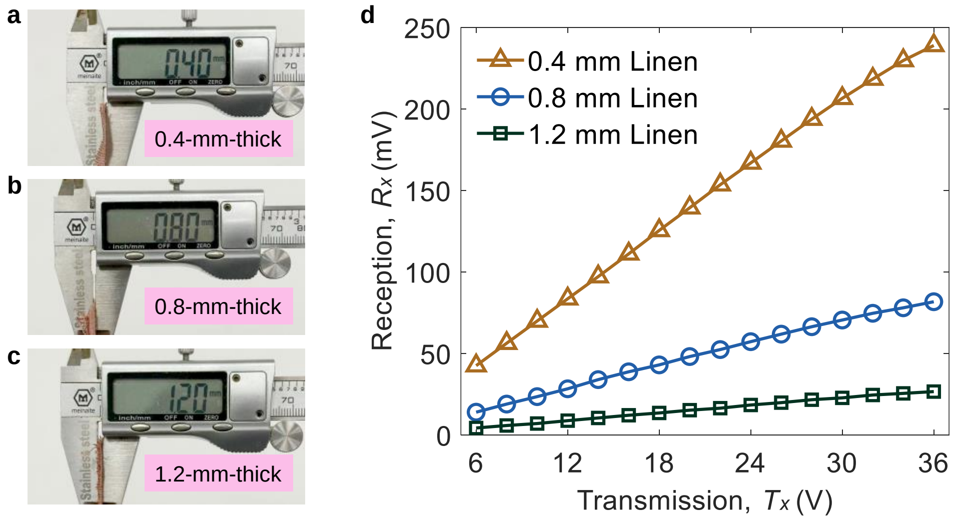

Supplementary Fig. 15 | Experimental analysis of the impact of textile substrate thickness on wave propagation in SonoTextiles. (a-c) Images of three linen textile substrates with different thicknesses, made from the same material and weaving. (d) Input-output characterization results of the SISO SonoTextiles system using these substrates, under the same experimental conditions (101 kHz, 12 stitches per 5 cm). The effect of substrate thickness on the R_x signal amplitude is evident. A thicker textile substrate absorbs more acoustic wave energy during propagation, leading to a lower R_x signal amplitude.

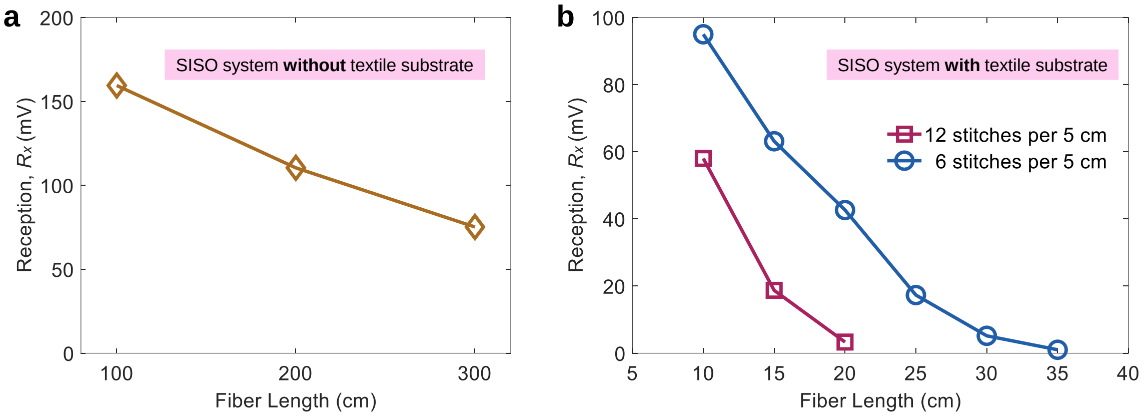

Supplementary Fig. 16 | Evaluation of wave travel distances in SonoTextiles with and without a textile substrate, assessed through the R_x amplitude. (a) Reception R_x amplitude versus fibre length for a SISO SonoTextile without textile substrate. It can be observed that without the textile substrate, the R_x amplitude remains as high as 75 mV even with a fibre length of up to 300 cm, though it shows a slight decrease compared to the 100 cm fibre length. This indicates that acoustic waves can propagate over long distances along the fibre. (b) Reception R_x amplitude versus fibre length for a SISO SonoTextile with textile substrate in different weaving conditions. Under the same stitch density, the R_x amplitude exhibits noticeable attenuation as the fibre length increases. With 6 stitches per 5 cm, the attenuation is less pronounced compared to 12 stitches per 5 cm. As a result, the former achieves an effective propagation distance of about 35 cm, while the latter achieves about 20 cm. The textile and PZT used were the same as those in Fig. 2. In both cases, the operating signal was a 101 kHz continuous wave with a peak-to-peak voltage of 36 V, and the SonoTextiles are not subjected to any external stimuli.

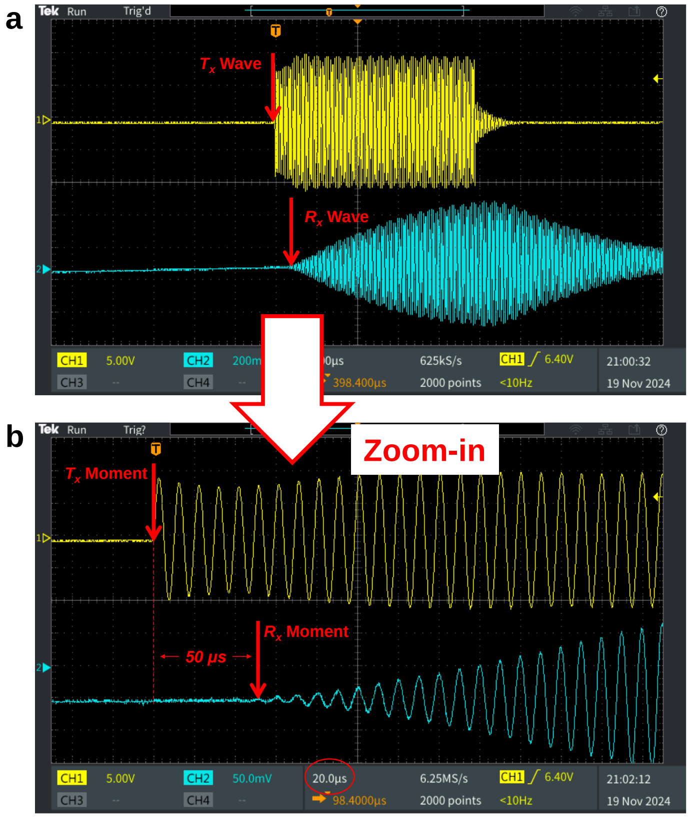

Supplementary Fig. 17 | Observation of wave propagation delay in the single-input single-output SonoTextiles using a pulse signal. (a) Screenshot of the oscilloscope, with two channels displaying the T_x and R_x waves, respectively. The length of the fibre in this case was 15 cm. The pulse signal emitted by the T_x PZT exhibits a noticeable delay before reaching the R_x PZT. The increasing R_x amplitude is due to response delays from mechanical damping and inertia, with energy building up as the system oscillates. The decay follows as the system dissipates energy through damping and the absence of continuous excitation. This phenomenon occurs with a pulse signal but not with a continuous wave. (b) Zoomed-in view of the above screenshot, showing a clear delay of 50 μs. This demonstrates the rapid signal propagation in SonoTextiles and highlights its fast response time.

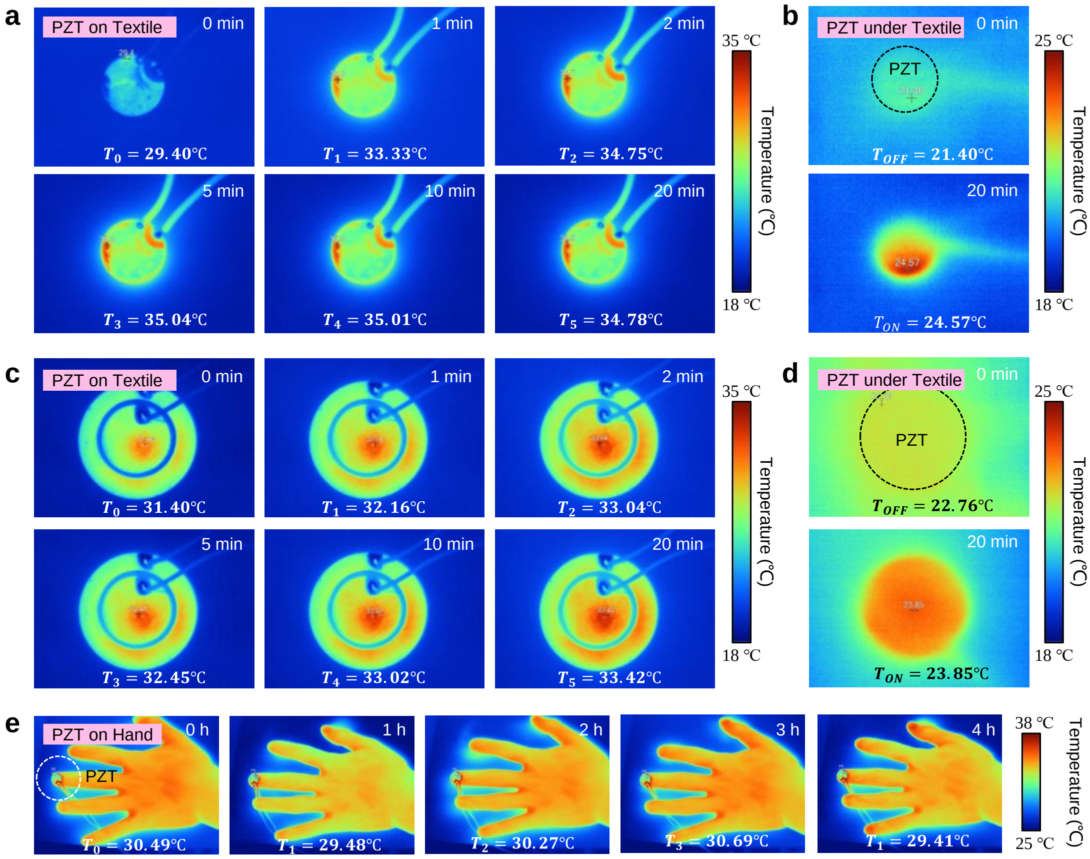

Supplementary Fig. 18 | Temperature variation during continuous operation of two types of PZTs. (a) Infrared camera recorded results for the 10-mm-diameter PZT placed on a nylon textile show operation at 181 kHz with a 20 Vpp input, causing a temperature rise from 29 °C to 35 °C within 2 minutes, stabilizing thereafter. (b) Results for the 10-mm-diameter PZT placed under the textile, with all other settings unchanged, show that after 20 minutes, the textile did not experience significant temperature rise due to PZT operation. (c) Results for the 27-mm-diameter PZT placed on a nylon textile show operation at 101 kHz with 20 Vpp input, showing a modest temperature increase from 31 °C to 33 °C over 2 minutes before stabilizing. (d) Results for the 27-mm-diameter PZT placed under the textile, with all other settings unchanged, indicate that after 20 minutes, the textile did not experience a significant temperature rise. (e) Results for the 10-mm-diameter PZT placed on the hand during 4-hours of continuous activation. The temperature shows no significant increase and remains relatively stable. The ambient temperature was around 22 °C. These results confirm that the PZTs in SonoTextiles do not generate significant heat under normal conditions.

Supplementary References

1. López, J. A. H., Oballe-Peinado, Ó. & Sánchez-Durán, J. A. A Proposal to Eliminate the Impact of Crosstalk on Resistive Sensor Array Readouts. *IEEE Sens. J.* **20**, 13461–13470 (2020).
2. Hannigan, B. C., Cuthbert, T. J., Ahmadizadeh, C. & Menon, C. Distributed sensing along fibers for smart clothing. *Sci. Adv.* **10**, eadj9708 (2024).
3. Saxena, R. S., Saini, N. K. & Bhan, R. K. Analysis of Crosstalk in Networked Arrays of Resistive Sensors. *IEEE Sens. J.* **11**, 920–924 (2011).
4. Giurgiutiu, V. Chapter 5 - Elastic Waves. in *Structural Health Monitoring with Piezoelectric Wafer Active Sensors (Second Edition)* 199–292 (Academic Press, Oxford, 2014). doi:10.1016/B978-0-12-418691-0.00005-8.
5. Cai, K., Mankar, S., Maslova, A., Ajiri, T. & Yotoriyama, T. Amplified piezoelectrically actuated on-chip flow switching for a rapid and stable microfluidic fluorescence activated cell sorter. *RSC Adv.* **10**, 40395–40405 (2020).
