# Supplementary material for: A smart acoustic textile for health monitoring
Source: Nat Electron. 2025 May 19;8(6):485–95. doi: 10.1038/s41928-025-01386-2 (PMC12202489; doi:10.1038/s41928-025-01386-2)
Supplement: Supplementary file 2 — Reporting Summary [file 41928_2025_1386_MOESM2_ESM.pdf]

nature portfolio

Corresponding author(s): Daniel Ahmed
Last updated by author(s): Mar 10, 2025

Reporting Summary

Nature Portfolio wishes to improve the reproducibility of the work that we publish. This form provides structure for consistency and transparency in reporting. For further information on Nature Portfolio policies, see our Editorial Policies and the Editorial Policy Checklist.

Statistics

For all statistical analyses, confirm that the following items are present in the figure legend, table legend, main text, or Methods section.

n/a	Confirmed	
☐	☒	The exact sample size (*n*) for each experimental group/condition, given as a discrete number and unit of measurement
☐	☒	A statement on whether measurements were taken from distinct samples or whether the same sample was measured repeatedly
☒	☐	The statistical test(s) used AND whether they are one- or two-sided<br>*Only common tests should be described solely by name; describe more complex techniques in the Methods section.*
☒	☐	A description of all covariates tested
☐	☒	A description of any assumptions or corrections, such as tests of normality and adjustment for multiple comparisons
☐	☒	A full description of the statistical parameters including central tendency (e.g. means) or other basic estimates (e.g. regression coefficient) AND variation (e.g. standard deviation) or associated estimates of uncertainty (e.g. confidence intervals)
☒	☐	For null hypothesis testing, the test statistic (e.g. *F*, *t*, *r*) with confidence intervals, effect sizes, degrees of freedom and *P* value noted<br>*Give P values as exact values whenever suitable.*
☒	☐	For Bayesian analysis, information on the choice of priors and Markov chain Monte Carlo settings
☒	☐	For hierarchical and complex designs, identification of the appropriate level for tests and full reporting of outcomes
☒	☐	Estimates of effect sizes (e.g. Cohen's *d*, Pearson's *r*), indicating how they were calculated

Our web collection on statistics for biologists contains articles on many of the points above.

Software and code

Policy information about availability of computer code

Data collection	The observation and recording of the received signals are performed by a digital oscilloscope (TBS 2000, Tektronix), which can display the signal waveform and FFT results in real-time and store the waveform on a USB device. A Sony Alpha 7 III camera was used to document the images of the relevant experiments.
Data analysis	The data processing of the received signal, which includes FFT-obtained single-sided amplitude spectra and time-frequency spectrograms, is primarily performed through post-processing using computer software (MATLAB 2024a, MathWorks).

For manuscripts utilizing custom algorithms or software that are central to the research but not yet described in published literature, software must be made available to editors and reviewers. We strongly encourage code deposition in a community repository (e.g. GitHub). See the Nature Portfolio guidelines for submitting code & software for further information.

Data

Policy information about availability of data

All manuscripts must include a data availability statement. This statement should provide the following information, where applicable:

- Accession codes, unique identifiers, or web links for publicly available datasets
- A description of any restrictions on data availability
- For clinical datasets or third party data, please ensure that the statement adheres to our policy

The datasets supporting the findings of this study are provided in the main text and supplementary materials. The raw data underlying the analysis can be obtained from the corresponding author upon reasonable request.

Human research participants

Policy information about studies involving human research participants and Sex and Gender in Research.

Reporting on sex and gender	Data from respiratory monitoring and hand gesture recognition from one male subject.
Population characteristics	We collected data from a healthy male human subject under the age of 30 years.
Recruitment	We recruited one participant from among the researchers involved in developing the smart acoustic textiles to test the initial feasibility of the technology. Participant selection is irrelevant to the results, as the experiments evaluate the system's functionality rather than individual characteristics.
Ethics oversight	This study was approved by the ETH Zurich Ethics Commission (No. EK-2024-E-18). All procedures were conducted in accordance with the approved guidelines and regulations. Informed consent was obtained from the participant prior to the participant's involvement in the study.

Note that full information on the approval of the study protocol must also be provided in the manuscript.

Field-specific reporting

Please select the one below that is the best fit for your research. If you are not sure, read the appropriate sections before making your selection.

☒ Life sciences ☐ Behavioural & social sciences ☐ Ecological, evolutionary & environmental sciences

For a reference copy of the document with all sections, see nature.com/documents/nr-reporting-summary-flat.pdf

Life sciences study design

All studies must disclose on these points even when the disclosure is negative.

Sample size	We collected data from a healthy male human subject under the age of 30 years. Participant selection is irrelevant to the results, as the experiments evaluate the system's functionality rather than individual characteristics.
Data exclusions	No data acquired for quantitative analysis were excluded.
Replication	A minimum of three independent experiments were conducted for the majority of conditions, and all attempts at replication were successful.
Randomization	This study conducts a systematic work on wearable electronics, primarily aiming to achieve a specific function, without involving the issue of random allocation of a large number of samples or participants. As such, the issue of covariate control is not applicable to this study, as the experiments were not designed to compare different groups or conditions. The primary objective was to assess the functionality of the technology, and no additional covariates were considered in the analysis.
Blinding	A blind approach was not feasible in this experiment because the procedures were observable. However, all samples were collected and analyzed under consistent external conditions.

Reporting for specific materials, systems and methods

We require information from authors about some types of materials, experimental systems and methods used in many studies. Here, indicate whether each material, system or method listed is relevant to your study. If you are not sure if a list item applies to your research, read the appropriate section before selecting a response.

Materials & experimental systems

n/a	Involved in the study	
☒	☐	Antibodies
☒	☐	Eukaryotic cell lines
☒	☐	Palaeontology and archaeology
☒	☐	Animals and other organisms
☒	☐	Clinical data
☒	☐	Dual use research of concern

Methods

n/a	Involved in the study	
☒	☐	ChIP-seq
☒	☐	Flow cytometry
☒	☐	MRI-based neuroimaging
